# Sparsity-Penalized Stacked Denoising Autoencoders for Imputing Single-Cell RNA-seq Data

**DOI:** 10.3390/genes11050532

**Published:** 2020-05-11

**Authors:** Weilai Chi, Minghua Deng

**Affiliations:** 1 Center for Quantitative Biology, Academy for Advanced Interdisciplinary Studies, Peking University, Beijing 100871, China; wlchi@pku.edu.cn; 2 School of Mathematical Sciences, Peking University, Beijing 100871, China; 3 Center for Statistical Science, Peking University, Beijing 100871, China

**Keywords:** single-cell RNA-seq, imputation, stacked denoising autoencoders, sparsity penalization

## Abstract

Single-cell RNA-seq (scRNA-seq) is quite prevalent in studying transcriptomes, but it suffers from excessive zeros, some of which are true, but others are false. False zeros, which can be seen as missing data, obstruct the downstream analysis of single-cell RNA-seq data. How to distinguish true zeros from false ones is the key point of this problem. Here, we propose sparsity-penalized stacked denoising autoencoders (scSDAEs) to impute scRNA-seq data. scSDAEs adopt stacked denoising autoencoders with a sparsity penalty, as well as a layer-wise pretraining procedure to improve model fitting. scSDAEs can capture nonlinear relationships among the data and incorporate information about the observed zeros. We tested the imputation efficiency of scSDAEs on recovering the true values of gene expression and helping downstream analysis. First, we show that scSDAE can recover the true values and the sample–sample correlations of bulk sequencing data with simulated noise. Next, we demonstrate that scSDAEs accurately impute RNA mixture dataset with different dilutions, spike-in RNA concentrations affected by technical zeros, and improves the consistency of RNA and protein levels in CITE-seq data. Finally, we show that scSDAEs can help downstream clustering analysis. In this study, we develop a deep learning-based method, scSDAE, to impute single-cell RNA-seq affected by technical zeros. Furthermore, we show that scSDAEs can recover the true values, to some extent, and help downstream analysis.

## 1. Introduction

Single-cell RNA sequencing (scRNA-seq) has revolutionized the study of transcriptomes since the earliest scRNA-seq technique was developed by Tang in 2009 [1]. scRNA-seq techniques have been applied to many research areas, such as neurons [2], immunology [3], and cancer [4] because they can study the whole transcriptome of each individual cell in high resolution. A large volume of scRNA-seq datasets has been produced, including cell atlases for humans [5] and mice [6,7]. However, different from bulk sequencing data, single-cell expression matrices contain an additional abundance of zeros because of biological and technical factors. What heavily troubles researchers are the technical zeros. Technical zeros are false zeros resulting from measuring errors as distinct from true zeros for genes not expressed. If gene expression is relatively high in some cells, but not in others of the same type [8,9], technical zeros are likely to happen. Technical zeros are quite prevalent in scRNA-seq data for the low capture of RNA molecules and relatively shallow sequencing depth. Since false zeros severely obstruct the downstream analysis of data, it is very important to distinguish technical zeros from true zeros and impute the true values of the missing data. 

Several methods aim at solving the missing data imputation problem in single-cell RNA-seq data. MAGIC [10] is the first method developed to solve this problem. MAGIC first constructs a Markov affinity-based matrix from a nearest-neighbor graph and fills in the missing transcripts via data diffusion. Diffusion time for the Markov affinity matrix is so hard to determine that it is easy for MAGIC to over-impute the expression matrix. Furthermore, MAGIC imputes all values in the expression matrix without distinguishing zero values from non-zero values. scImpute [11] first fits a Gamma–Normal mixture model to calculate the missing probabilities for each gene in each cell and then finds the positions which need imputing and which can be seen as a reference to select similar cells by cutting a missing probability threshold. Finally, scImpute imputes the expression of genes needing imputing by borrowing information from the same gene’s expression in similar cells learned from reference genes by non-negative least squares (NNLSs) regression. scImpute implicitly assumes linear relationships among cells, which may not hold for actual situations. SAVER [12] models the UMI [13] counts with the Gamma–Poisson model, the parameters of which are estimated with an empirical Bayes-like technique. Moreover, the missing values are imputed with the posterior mean of the normalized true expression. SAVER is slow and consumes much memory owing to its LASSO regression for each gene. ALAR [14] and scRMD [15] both assume the expression matrix is low-rank and capture linear relationships in the data matrix. ALAR computes a low-rank approximation of the expression matrix using singular vector decomposition (SVD), while scRMD adopts robust matrix decomposition to impute the zero values.

The autoencoders are manifold learning algorithms assuming that high-dimensional data actually lie on a low-dimensional manifold. Specifically, autoencoders first compress high-dimensional data to a low-dimensional hidden space and then reconstruct it using artificial neural networks. For their abilities to regenerate and denoise the input data, autoencoders are often utilized to do dimension reduction and missing data imputation. The loss of classical autoencoders is the reconstruction error on all features, while for imputing missing data, the reconstruction error on the non-missing features is often used. There are several pure autoencoder-based methods developed to impute missing data in scRNA-seq data. SAUCIE [16] uses the classical reconstruction loss on all values, while AutoImpute [17] and LATE [18] adopt reconstruction loss only on non-zero values. SAUCIE is a multitasking model based on regularized autoencoders itself and degenerates to an imputation model when no batch information and clustering task are provided. SAUCIE replaces all the data with the neural network output, which implements both imputation and denoising on the data. AutoImpute is based on shallow autoencoders with one hidden layer. LATE adopts deep autoencoders and also introduces transfer learning to initialize the weights of the networks to improve training. However, the classical loss cannot treat the zero-values and the non-zero values differently, while loss only on non-zero values loses the information of the true zero values. DCA [19] (deep count autoencoder) and scVI [20] (single-cell variational inference) are methods that combine statistical modeling with deep neural networks. DCA exchanges the regular output of the autoencoder for the parameters of the distribution of a noise model, such as ZINB (zero-inflated negative binomial) distribution, and adopts the log-likelihood of the noise model as the loss to train a deep autoencoder-based model. ZINB noise model-based DCA imputes all missing values directly with the mean of the output NB distribution without considering the estimated zero-inflated probability, which may cause over-imputation. scVI also uses ZINB distribution to model the count matrix, but it incorporates a deep generative model, deep variational autoencoders [21] to build the generative and inference architecture. scVI can not only impute missing values but also cluster the data and do differential expression analysis. There are also other imputation methods based on deep learning, such as DeepImpute [22] based on sub-neural networks and GraphSCI [23] based on graph convolutional autoencoders.

Here, we propose sparsity-penalized stacked denoising autoencoders (scSDAEs), a method that adopts layer-wise pretrained stacked denoising autoencoders as the imputation framework and the mixture of mean squared error (MSE) and sparse penalty as the loss to impute the missing data in scRNA-seq data. Our method has three advantages. First, it has weak assumptions on data distribution. Second, it incorporates the information of zeros but treats them differently from non-zero data. Third, layer-wise pretrained stacked denoising autoencoders improve the fitting ability of the model and enable us to fit the model better without filtering genes out, especially on small-size datasets. We compare the recovery ability of our method with three state-of-the-art methods, including MAGIC, scImpute, DCA, and other autoencoder-based methods, such as SAUCIE, SDAE and SDAE0.

SAUCIE imputes and denoises all the values in the expression matrix without distinguishing zero values and non-zero ones. Because autoencoder-based methods assume the data lie on a low-dimensional manifold, replacing all the values makes the expression matrices lose their biological heterogeneity and induces many false signals. SAUCIE sometimes imputes all the cells into similar vectors with little variation (Figures S3 and S5b). To make effective measurements and better demonstrate the effectiveness of the mixture loss of scSDAE, we only test SAUCIE on simulation data and compare two other SDAE models that only impute zero values in real data analysis. The two SDAE models share the network architecture with scSDAE but differ in the loss function. The first one with classical loss is denoted as SDAE, and the second one with only the loss of non-missing features is denoted as SDAE0. To be fair, the layer-wise pretraining strategy was also applied to SDAE and SDAE0. 

## 2. Materials and Methods 

### 2.1. Data Collection and Pre-Processing

First, we removed the cells in which no genes expressed and the genes that were expressed in no cells. After filtering, tens of thousands of genes still remained in the expression matrix, which needed handling by relatively deep neural networks. Then, we normalized the data by library size and took the logarithm of the library-size-normalized expression data with Pesudocount 1:

(1) $$x=\log\left(\frac{count}{\mathrm{sum}\left(count\right)}\times 10^{6}+1\;\right)$$

The raw count vector of a cell with n genes expressing is denoted as count, and the normalized expression vector of a cell is denoted as $x=\left(x_{1},\;\cdots x_{n}\right)$. We used cell-based autoencoders, the input of which was the normalized expression vector of a cell.

### 2.2. scSDAE Model Structure and Training Procedure

#### 2.2.1. Autoencoders

An autoencoder is an artificial neural network trained to reconstruct the input data itself. It usually consists of three layers: an input layer, a hidden layer, and an output layer. The output layer has the same dimension as the input layer, while the hidden layer is of much lower dimension in order to facilitate leaning a low dimensional representation of the input data. Specifically, an autoencoder maps the input data to the hidden layer z through an encoder function $z=f_{\omega }\left(x\right)$ and reconstructs the output data $x^{\prime }$ from the hidden layer through a decoder function $x^{\prime }=g_{\omega^{\prime }}\left(z\right)$. $f_{\omega }\left(\cdot \right)$ and $g_{\omega^{\prime }}\left(\cdot \right)$ are neural network layers with activation functions. By minimizing reconstruction loss between input data and output data, such as MSE loss, the autoencoder learns the weights $\left\{\omega ,\omega^{\prime }\right\}$ of the neural networks without any extra information.

#### 2.2.2. Denoising Autoencoders (DAE)

Vincent et al. [24] developed denoising autoencoders (DAE) to reconstruct the input x from a corrupted version $\tilde{x}$ of it, which is a more challenging task than the basic autoencoders. The encoder and decoder can be formulated as $z=f_{\omega }\left(\tilde{x}\right),\;x^{\prime }=g_{\omega^{\prime }}\left(z\right)$, and the loss function is still the reconstruction error between the output $x^{\prime }$ and the uncorrupted input x:

(2) $$L_{SDAE}\left(x,\;x^{\prime }\right)=\frac{1}{n}||x-x^{\prime }||{}^{2}$$

$\tilde{x}$ is corrupted with some noise, such as 0-1 mask, isotropic Gaussian noise, and salt-and-pepper noise [24]. In our paper, we randomly mask some observed input features to zeros to get the corrupted ones. Denoising autoencoders are supposed to learn more stable and robust representations.

#### 2.2.3. Sparsity-Penalized Stacked Denoising Autoencoders (scSDAEs) 

We used stacked denoising autoencoders (SDAEs) [24] as the imputation framework for scRNA-seq data on the assumption that the expression data lie on a low-dimensional manifold. When the data structure is much more complex, deeper architectures, such as SDAEs, are necessary. SDAEs stack several autoencoders by taking the hidden layer of the previous autoencoder as the input layer of the next autoencoder. SDAEs compress the data layer by layer in the encoders and reconstructs the output layer by layer in the decoders. SDAE with k encoder layers and k decoder layers is like

(3) $$z=f_{\omega_{k}}\circ \cdots \circ f_{\omega_{1}}\left(x\right)\;x^{\prime }=g_{\omega_{1}^{\prime }}\circ \cdots \circ g_{\omega_{k}^{\prime }}\left(z\right)$$

The structure of this neural network is symmetrical where the input of the ith encoder layer $f_{\omega_{i}}\left(\cdot \right)$ has the same dimension as the output of the ith decoder layer $g_{\omega_{i}^{\prime }}\left(\cdot \right)$. 

We adopt the following mixture loss function for scSDAE:

(4) $$L_{scSDAE}\left(x,\;x^{\prime }\right)=\frac{1}{\#\{j:x_{j}>0\}}\underset{x_{j}>0}{\sum }{\left(x_{j}-x_{j}\prime \right)}^{2}+\alpha \frac{1}{\#\left\{j:x_{j}=0\right\}}\underset{x_{j}=0}{\sum }\left|{x^{\prime }}_{j}\right|$$

**Figure 1:**
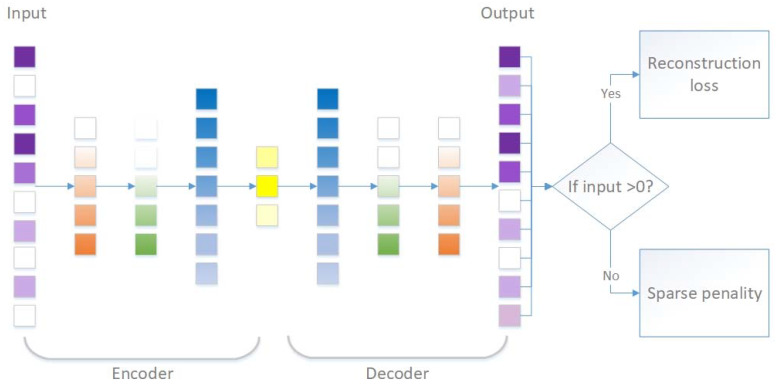
Schematic of scSDAE to impute scRNA-seq data. The figure shows the stacked autoencoders with a mixture of reconstruction loss on non-zero values and sparse penalty on zero values. The shades of the boxes represent the values. The darker the colors, the bigger the values. Additionally, white boxes represent zero values. The input is the normalized expression vector of a cell (purple boxes), and the output is of the same dimension as input (also purple boxes with less zeros). The yellow boxes depict the bottleneck layer, while blue boxes, green boxes, and orange boxes indicate the hidden layers of the stacked autoencoders.

The first part of the loss function is the same as others [17,25] to minimize the reconstruction error of the observed values. The second part is the L1 penalty, which was shown to be efficient to constrain the sparsity of imputed values in matrix completion [26]. The L1 penalty aims at two things. On the one hand, it is designed to penalize the number of non-zero imputed values in order to preserve the true zeros. On the other hand, it can shrink the imputed values weakly since small values can easily be missing [27,28]. The schematic of our imputing framework is shown in Figure 1.

The word “denoising” reflects in the training strategy of scSDAE, which is from Vincent et al. [24]. The training strategy is important for scSDAE, especially when the dataset size is relatively small because deep unsupervised neural networks are quite difficult to train. First, scSDAE is trained layer by layer. Each layer of scSDAE is regarded as a denoising autoencoder in which the input is predicted from the previous layer and then corrupted; the aim is to recover the uncorrupted version of it. Once the autoencoder has been built and trained, it will henceforth be applied to the uncorrupted input to get the hidden layer. Then the hidden layer will be taken as the input of the next layer to repeat the training of the denoising autoencoders until all the layers of scSDAE have been pretrained. Finally, the trained autoencoders are stacked together to form a deep neural network without corruption, which will be fine-tuned by minimizing the reconstruction loss of the original input. We only impute the zero values in the expression matrix, so we add a mask matrix to keep the non-zero values. We will show more details in Algorithm 1.
**Algorithm 1.** Sparsity penalized stacked denoising autoencoders for single cell RNA-seq data (scSDAE).**Input**: normalized expression vector $x=\left(x_{1},\cdots x_{n}\right)$, network layer width vector $dim=\left(n,d_{1},\cdots d_{k}\right)$ with $d_{k}$ denoted the bottleneck layer width**Output**: imputed expression vector $x_{imputed}$**for**i in {1,⋯k}
**do**
          **if**
i==1
**do**
               corrupt x into $\tilde{x}$ with noise, build neural network $NN_{1}\left(n\to d_{1}\to n\right)$: $x\prime =g_{\omega_{1}^{\prime }}(f_{\omega_{1}}\left(\tilde{x}\right))$ and train $NN_{1}$ to minimize $L_{scSDAE}\left(x,\;x\prime \right)$; predict $h_{1}=f_{\omega_{1}}\left(x\right)$
          **else do**
               corrupt $h_{i-1}$ into ${\tilde{h}}_{i-1}$ with noise, build neural network $NN_{i}\left(d_{i-1}\to d_{i}\to d_{i-1}\right)$: $h_{i-1}^{\prime }=g_{\omega_{i}^{\prime }}(f_{\omega_{i}}\left({\tilde{h}}_{i-1}\right))$ and train $NN_{i}$ to minimize formula $||h_{i-1}-h_{i-1}^{\prime }||{}^{2}/d_{i-1}$
**if**i<k**do**predict $h_{i}=f_{\omega_{i}}\left(h_{i-1}\right)$
**end for**build neural network NN: $x^{\prime }=g_{\omega_{1}^{\prime }}\circ \cdots \circ g_{\omega_{k}^{\prime }}\left(f_{\omega_{k}}\circ \cdots \circ f_{\omega_{1}}\left(x\right)\right)$ and train NN to minimize $L_{scSDAE}\left(x,\;x\prime \right)$
predict $x_{out}=g_{\omega_{1}^{\prime }}\circ \cdots \circ g_{\omega_{k}^{\prime }}\left(f_{\omega_{k}}\circ \cdots \circ f_{\omega_{1}}\left(x\right)\right)$
calculate the mask vector m: $m_{j}=I\left(x_{j}>0\right),\;j=1\cdots n\;$ (I(·) is the indicator function.) calculate the output $x_{imputed}=x_{out}*\left(1-m\right)+x*m$ (∗ denotes element-wise multiplication.)

### 2.3. Parameter Setting and Implementation 

The encoder network is set as a fully connected multilayer perceptron (MLP) with dimensions n-500-500-2000-10 for all datasets. In addition, the decoder network is an MLP with dimensions 10-2000-500-500-n. While the output and the embedding layer are not activated by any activation functions, all other layers are activated by ReLU nonlinear function. Parameter α for the simulated dataset from bulk data is set to be 0.001, for there are few zeros in the bulk data itself, and the simulated zeros are false zeros that should not provide information for the loss function. Parameter α for other experiments is set to be 1.0 after a grid search. We have tested parameter α several times, out of which 1.0 can get a satisfactory performance on most datasets in our paper. We suggest people choose α from (0.1, 1) according to actual conditions. The corruption rate during the pretraining of each input layer is set to be 0.2, and we use MSE loss for each internal pretraining autoencoder and a mixture of MSE and sparse penalty for the output layer. Adam optimizer [29] with default parameters and a minibatch size of 256 is used. Each layer is pretrained for 1000 iterations. The total network is fine-tuned for 2000 iterations. scSDAE is implemented with Keras library.

The computations and analyses were carried out using R version 3.6.1 (R Core Team, Vienna, Austria) and Python version 3.7.3 (Python Software Foundation, Wilmington, DE, USA). The R package “splatter” (v.1.8.0) for simulation and the packages for normalization were installed from Bioconductor. The normalization packages, including scran (v.1.12.1), Linnorm (v.2.8.0), and DESeq2 (v.1.24.0) were performed with default settings, while for scone (v.1.8.0), we set the maximum number of unwanted variation components to zero. The Python version of the MAGIC package (v.1.5.99) was installed with pip, and the default parameters were used. The scImpute R package (v.0.0.8) was downloaded from GitHub (https://github.com/Vivianstats/scImpute), and the default parameters were used. The DCA Python package (v.0.2.2) was installed with pip. The hidden_size and batch_size parameters were set the same as those of scSDAE, and other parameters were set as default. The SAUCIE package was downloaded from GitHub (https://github.com/KrishnaswamyLab/SAUCIE), and the default parameters were used. The Seurat R package (v.3.0) for clustering analysis was downloaded from R cran (https://CRAN.R-project.org/package=Seurat). We used the first 30 principal components to do clustering analysis and visualization and set the resolution parameter to make the number of communities as close as possible to the given cluster number. Please refer to the code to implement scSDAE with Python in Appendix A.

## 3. Results

### 3.1. scSDAE Recovers Gene Expression Affected by Simulated Missing Values

**Figure 2:**
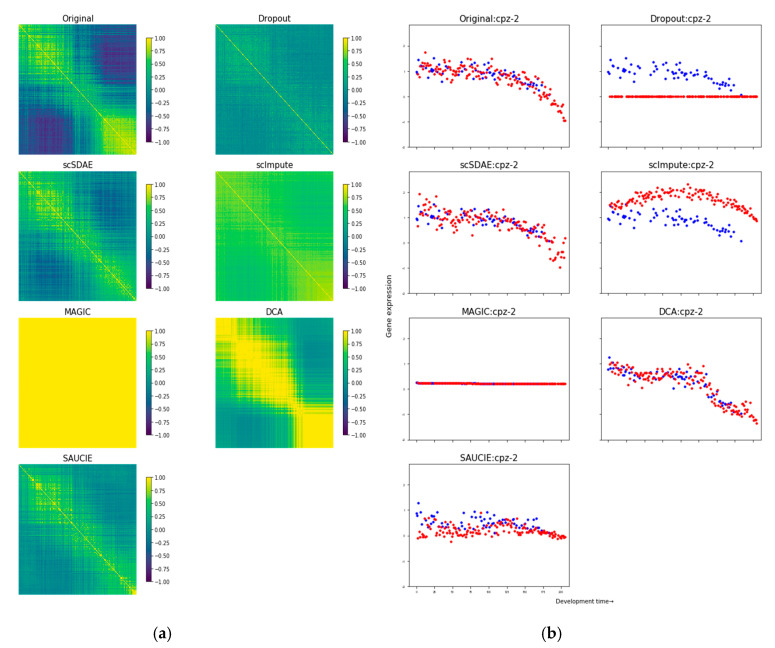
Performance comparison of simulated data. (**a**) The heatmaps of sample-to-sample correlation matrices show scSDAE best recovered sample developmental trajectory affected by simulated missing values. The samples are ordered by developmental time. So as is shown in the original data and scSDAE, the correlations around diagonal should be high, but correlations off the diagonal should not. (**b**) Scatter plots of gene expression trajectory show that scSDAE best recovered gene expression affected by simulated missing values for gene *cpz-2*. Scatter plots of the expression trajectory of randomly selected gene *cpz-2* from the original bulk data, simulated missing data, and expression data imputed after different imputation methods are shown. The horizontal axis represents the development time of the samples. The vertical axis stands for the gene expression value. Each point represents a sample. The red points represent the values simulated to be zeros, and the blue ones represent the observed values.

We first downsampled the bulk data and regarded it as the golden standard to evaluate the performance of the imputation methods. To obtain the simulated data, we adopted the relationship observed by [28] that the logit of the probability of a gene being captured in the library linearly depends on the log of the gene’s true absolute molecule counts in the cell. The microarray expression profiles of 206 samples [30], representing the gene expression dynamics throughout a 12-hour period development of *Caenorhabditis elegans*, were utilized. We downsampled the bulk data to different sparsity levels by varying the coefficients of the logistic model and obtained simulated datasets containing 50%, 60%, 70%, 80%, and 90% zeros for ten times, respectively. The recovery of the expression values itself and sample-to-sample correlation matrices were assessed, for the sample-to-sample correlation matrix can reflect the sample developmental trajectory. First, we calculated the correlation matrix distance (CMD) [31] between the original matrix and the dropout/imputed matrix. A smaller CMD means the sample-to-sample correlation matrix is more similar to the original one. The mean and standard deviation of the CMDs among different replications are shown in Table 1. Then, we calculated the pairwise Pearson correlations between the bulk data and the dropout/imputed data. The mean and standard deviation of the correlations are shown in Table S1. scSDAE consistently obtained smaller CMDs and larger correlations than other imputation methods. In the meanwhile, only scSDAE got smaller CMDs than the raw data across all zero levels. We also selected the dataset containing 80% zeros as an example to analyze in detail. Figure 2a shows the heatmaps of the sample-to-sample correlation matrix using the top 200 most variable genes from the original bulk data. From Figure 2a, we can see that scSDAE recovered the sample-to-sample correlation matrix of the original bulk data best, while MAGIC, DCA, and scImpute over-imputed it and SAUCIE under-imputed it. The expression heatmaps of the top 200 most variable genes are in Figure S1. We also found that scSDAE best recaptured the expression dynamics during the development of some randomly selected genes (Figure 2b and Figure S2). 

### 3.2. Assessing the False Signals Induced by Different Imputation Methods

To test if false signals are induced by imputation methods, we followed the simulation strategy of [32]. We first applied imputation methods to the negative binomial simulation data to test if they introduced false gene–gene correlations. We independently simulated ten expression matrices. Each contained 1000 cells, equally spread across two cell-types, and 500 genes, with mean expression ranging from 10^3^–10^4^. Half of the genes were differentially expressed (DE) between the two cell-types, half were drawn independently. Spearman correlation with a conservative Bonferroni multiple testing correction (family-wise type I error rate *q* < 0.05) was used to identify significant gene–gene correlations. Figure S3a shows the gene-gene correlation heatmaps of one simulated dataset imputed by different imputation methods with genes sorted in descending order of expression. SAUCIE imputed all the cells into almost the same vector so that the heatmap of the gene–gene correlations cannot be drawn due to zero variance. Both MAGIC and DCA induced many spurious gene–gene correlations in non-differential genes, though they strengthened the gene–gene correlations in DE genes. scSDAE increased the sensitivity to detect gene-gene correlations among the lowly expressed DE genes but created spurious correlations mostly among lowly expressed non-differential genes, which was very similar to the result of [32]. We can see that there were also spurious correlations in no-imputed log-normalization data. The false signals in scSDAE could be explained by that slight biases and fake correlations induced when correcting for library-size in the presence of strong biological differences might be amplified by the imputation methods [32]. scImpute only slightly increased the gene–gene correlation detection with slight false signals. In Figure S3b, we varied the parameter α, which decides how many zeros to be imputed in our model. As expected, smaller α generated more false positive gene–gene correlations. The FDR and TPR were relatively stable when α was between 0.01 and 10. We also tested SAUCIE with different bottleneck layer hidden dimensions. The FDR of SAUCIE was close to 1, similar to that of DCA, which indicated severe spurious gene–gene correlations induced by SAUCIE and DCA. 

Next, we assessed the accuracy of different imputation methods to identify differentially expressed genes using simulated data with Splatter [33]. The simulated data contained 1000 cells split into 2–10 groups and 1000–5000 genes, with 1%–30% differentially expressed across the groups as in [32]. Four different levels of zero inflation and no zero inflation were considered (Table 2 in [32]). We ran a differential expression analysis between the groups using the non-parametric Kruskal–Wallis test [34] with a 5% FDR. First, we investigated the specificity and sensitivity of the imputation methods to detect DE genes. scSDAE and scImpute got slightly higher sensitivity and lower specificity than the unimputed data did (Figure S4A–D). However, SAUCIE and DCA got very low sensitivity and high specificity in most cases, with only several exceptions, for they might denoise cells into similar vectors with little differentially expressed genes. SAUCIE performed quite unstably when the dropout rate was high or the DE proportion was high. MAGIC showed a big difference among different conditions, even among different replications. Importantly, as for the trade-off between sensitivity and specificity, we considered the ROC curves. Figure S4E shows that the performance of scSDAE and scImpute is close to the unimputed data and better than all other methods. It is not surprising that no methods outperformed the unimputed data, for the result in [32] has validated that.

### 3.3. scSDAE Increases the Within-Group Similarity in RNA Mixture Dataset

**Figure 3:**
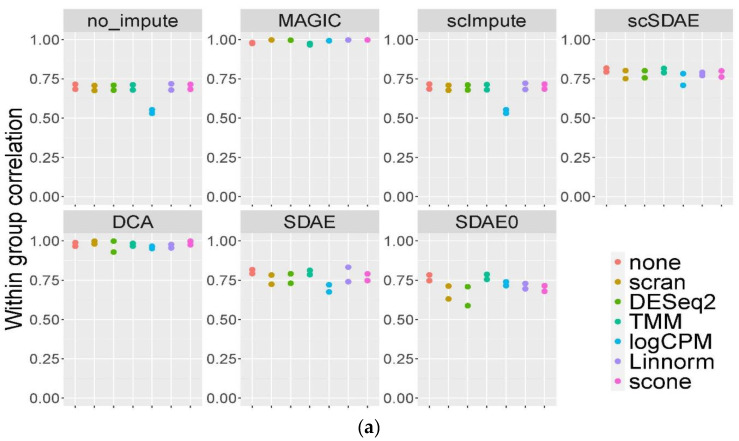

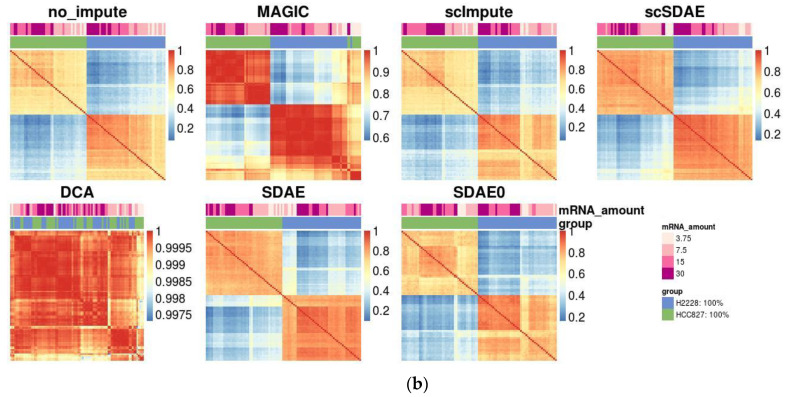
Comparisons of imputation methods using RNA mixture dataset. (**a**) Average Pearson correlation coefficients for cells within the same groups in the CEL-seq2 and Sort-seq datasets (n = 2) from different combinations of normalization and imputation methods. (**b**) Heatmaps of Pearson correlation coefficients for samples in the CEL-seq2 dataset that contained pure H2228 (n = 45) or HCC827 (n = 44) RNA obtained from different imputation methods after TMM normalization.

We adopted the RNA mixture dataset of [35] to validate the imputation accuracy of scSDAE. They sequenced mixtures of RNA from three cancer cell lines, including a dilution series to simulate variations in the RNA content of different cells. The RNA mixture contained 7 groups with different RNA mixture proportions sequenced by two plate-based (CEL-seq2 and SORT-seq) protocols. The known composition of RNA served as ground truth. To assess the effect of different normalization methods, we used five more normalization methods, besides log counts-per-million (logCPM) used in other experiments. The techniques developed primarily for bulk data, including the trimmed mean of M-values (TMM) [36] and DESeq2 [37], and others customized for scRNA-seq, such as scone [38], Linnorm [39], and scran [40], were incorporated. We evaluated the performance of the imputation methods using the Pearson correlation coefficients of normalized gene expression within each group for the RNA mixture data. Figure 3a shows the scatter plot of the within-group correlations from each normalization method and imputation method, where the two points in the same vertical line are from CEL-seq2 and SORT-seq, respectively. In general, scSDAE obtained higher intra-group correlations than the unimputed data and eliminated the difference among different normalization methods. scImpute hardly improved the within-group correlations, whereas MAGIC and DCA got intra-group correlations close to 1. Then we chose cells from two distinct groups (pure H2228 and HCC827) from the CEL-seq2 dataset and examined the correlations among samples. The heatmaps of Pearson correlations for chosen samples after TMM normalization and different imputations are shown in Figure 3b. In no-imputed data, when the amount of messenger RNA decreased, the sample correlations within the same group got lower, and spurious positive correlations between groups showed up. Although MAGIC and DCA had high within-group correlations, they introduced severe false signals with high between-group correlations. However, scSDAE and SDAE improved the correlations within-group, with low correlations between groups. scImpute and SDAE0 obtained a similar correlation structure with the data without imputation. PCA plots of the CEL-seq2 dataset (n = 340) after Linnorm normalization and different imputations are in Figure S5a. scSDAE not only improved the within-group purity but also kept the variations in the groups, which also validated the efficiency of scSDAE in recovering the RNA mixture data. 

### 3.4. scSDAE Accurately Recovers Spike-in RNA Concentrations Affected by Technical Zeros

**Figure 4:**
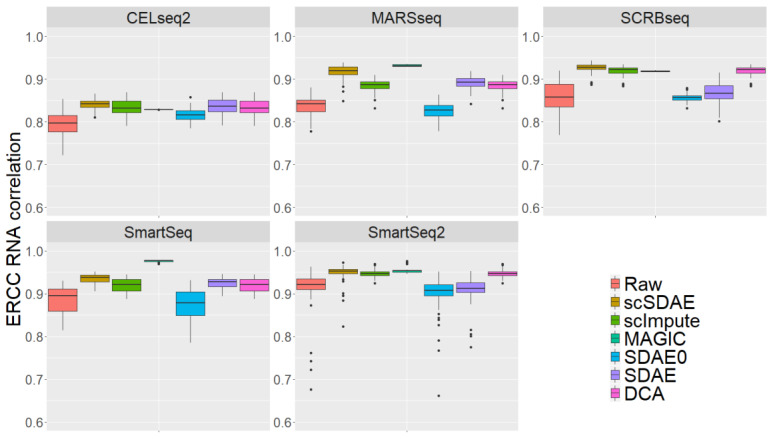
Boxplots of Pearson correlations between the imputed data and the known concentration of ERCC RNAs. Each panel represents a sequencing technique.

It is hard to directly evaluate the accuracy of imputation methods since the true expression of genes in real scRNA-seq data is unknown. However, spike-in control external RNAs with known concentrations can serve as a golden standard for comparison. Ziegenhain et al. [41] sequenced 583 mouse embryonic stem cells to evaluate six prominent scRNA-seq methods: CEL-seq2, Drop-seq, MARS-seq, SCRBseq, Smart-seq, and Smart-seq2. In addition, they spiked in 92 poly-adenylated synthetic RNA transcripts of known concentration designed by the External RNA Control Consortium (ERCC) [42] in 507 cells, except 67 Drop-seq sequencing cells. The ERCC RNAs have various lengths and GC content and cover a 220 concentration range [42], making them a good metric to evaluate the imputation methods. Since the sequencing values of the spike-in ERCC RNAs are influenced by cell library size, we studied imputation efficiency with Pearson correlations between the imputation values of each cell and the known concentration. We ran all the imputation methods separately on cells from each scRNA-seq method. Figure 4 shows the boxplots of the Pearson correlation. scSDAE achieved better performance than scImpute and DCA on all five scRNA-seq datasets, while MAGIC seemed to get slightly higher correlations than scSDAE on MARSseq, SmartSeq, and SmartSeq2. However, we calculated the Pearson correlations of ERCC RNAs for all cells after MAGIC imputation, the range of which was [0.9979, 1], implying that MAGIC replaced all cells with similar vectors and tended to over-imputed the expression data. scSDAE also defeated classical SDAE and SDAE0 without taking the information of zeros on all five scRNA-seq datasets. In conclusion, scSDAE can accurately recover spike-in RNA concentrations in scRNA-seq data from different sequencing techniques, even though the datasets only contain dozens of cells.

### 3.5. scSDAE Improves the Consistency of RNA and Protein Levels in CITE-seq Data

**Figure 5:**
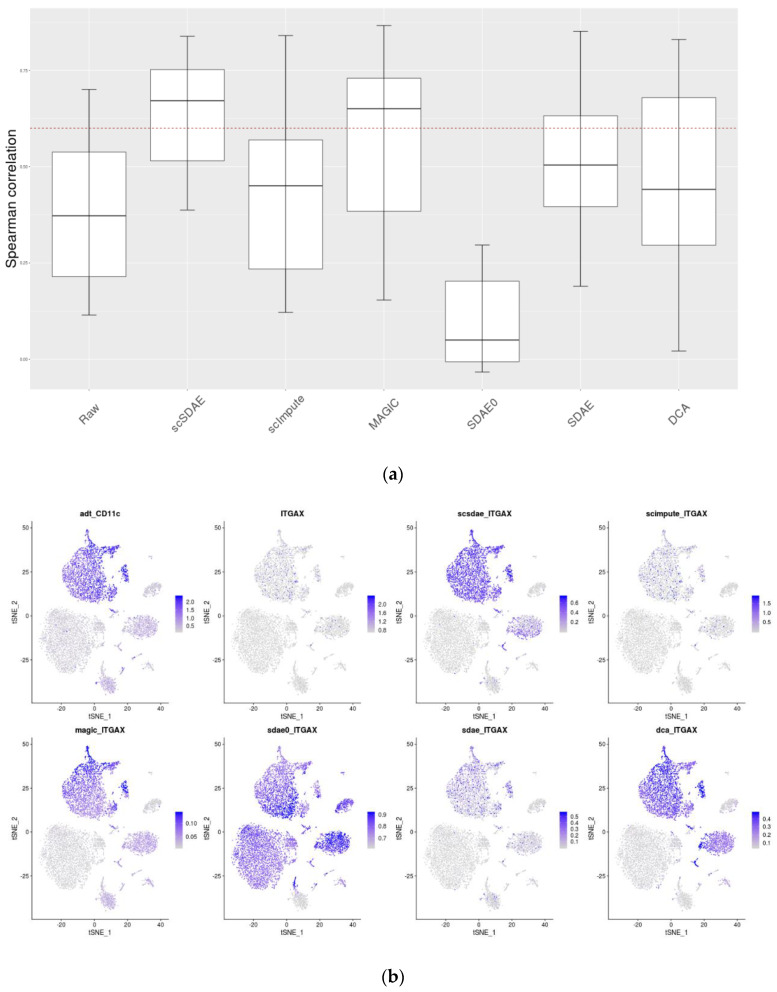
Consistency of RNA and protein levels in CITE-seq data. (**a**) Boxplot of the Spearman correlations of the protein-RNA pairs in CITE-seq data (The red dashed line represents *y* = 0.6). (**b**) Featureplot of the cells in CITE-seq data. TSNE visualizations of the cells are shown in the figure where each point represents a cell, and the colors of points indicate the expression levels of the protein and corresponding RNA of gene CD11c (*ITGAX*).

Here, we analyze a CITE-seq dataset [43] of 8617 cord blood mononuclear cells (CBMCs) containing two parts: the whole transcriptome expression and the expression of 8 surface proteins for every single cell. CITE-seq [43] can simultaneously measure the single-cell transcriptomes and the cell-surface protein expression level by sequencing. The significantly higher protein copy number in cells significantly reduces technical zeros in the protein expression profile, and protein expression can, therefore, serves as a silver standard to evaluate scRNA-seq data imputation methods. We evaluated the accuracy of different imputation methods by comparing their ability to improve the consistency of RNA and protein levels in CITE-seq data. Data analysis after imputation was carried out with Seurat [44]. First, we plotted the boxplot of the Spearman correlations (Figure 5a) and Pearson correlations (Figure S6) for protein–RNA pairs across all cells to evaluate the imputation methods. From Figure 5a, we can see that scSDAE obtained the highest median correlation for the protein-RNA pairs with the smallest variances and that only the median correlation of scSDAE and MAGIC was larger than 0.6. scSDAE0, which does not incorporate the information of zeros, performed the worst. Next, we compared the distribution of the proteins and the corresponding RNAs in the cells through a feature plot. In Figure 5b, tSNE [45] visualizations of cells are shown, the color shades of which indicate the expression levels of the protein and corresponding RNA of gene CD11c (ITGAX) within each cell. The level of consistency of the spatial distribution of protein and RNA expression in the figure indicates the efficiency of the methods. The feature plots of scSDAE, MAGIC, and DCA look like that of the protein. While SDAE0 over-imputed the expression heavily, scImpute and SDAE only imputed the expression slightly, indicating the efficiency of the mixture loss of scSDAE. Feature plots of other protein-RNA pairs are in Figures S7–S13.

### 3.6. scSDAE Can Help Improve Clustering Accuracy

We did a clustering analysis of five datasets with Seurat [44]. These datasets included the Kolodziejczyk data [46] with 704 cells and 38653 genes from 3 cell types, the Pollen [47] data with 301 cells and 23720 genes from 11 cell types, the Zeisel data [48] with 3005 cells and 19972 genes from 9 cell types, the Lake data [49] with 3042 cells and 25123 genes from 16 cell types, and the Patel [50] data with 430 cells and 5948 genes from 5 cell types. We benchmarked clustering accuracy on both imputed data and raw data with the labels of single cells, using the adjusted rand index (ARI) metric [51]. The results are shown in Table 2. We can see that the clustering accuracy on the data imputed by scSDAE was consistently better than that on the raw data and that scSDAE also outperformed other methods in most cases. DCA performed worst on several small-sized datasets, which might result from the underfitting of the deep neural networks. On the contrary, scSDAE performed very well, even on a small sample size, which demonstrated the power of layer-wise pretraining of the deep neural networks. We also plotted the two-dimensional UMAP [52] visualization of the Pollen dataset (Figures S14–S20). Pollen dataset captured 301 single cells from 11 populations, most of which were distinguishable populations from different tissues. However, there were also similar cell types, which represented three stages of neuronal differentiation, the germinal zone of the human cortex at gestational week (GW16), primary cells from the cortex at GW21 and GW21 cells further cultured for 3 weeks (GW21+3). According to the marker gene analysis in [47], GW21+3 mainly consisted of maturing neurons, while GW16 mainly contained radial glia and newborn neurons with little overlap from GW21+3. GW21 only contained 7 cells that might be the mixture of three cell types and, thus, hard to distinguish from GW16 and GW21+3. To test if the imputed data can better separate the similar cell types, we colored the points representing single cells from GW16, GW21, and GW21+3 with points of other cell types gray. We can see that only the UMAP plot of our method could distinguish GW16 and GW21+3 better than the raw data.

## 4. Discussion

**Figure 6:**
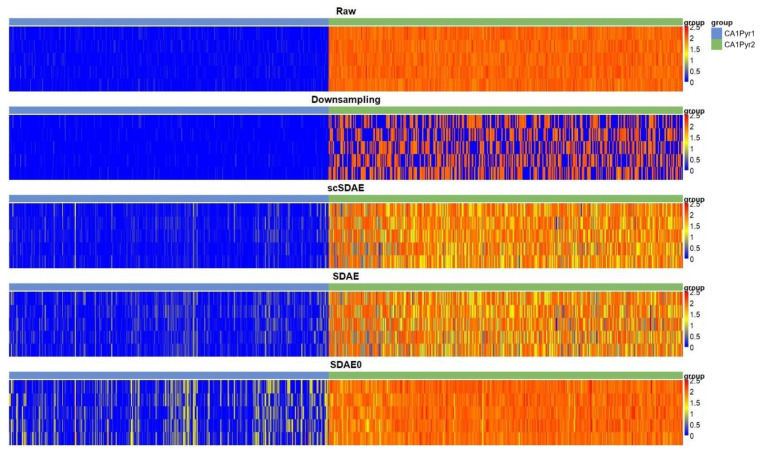
The heatmaps of five artificial genes with 50% zeros in the CA1Pyr1 and CA1Pyr2 groups are shown. The vertical axis represents the artificial genes, and the horizontal axis stands for the cells. True values in the CA1Pyr1 group are close to zero, while true values in the CA1Pyr2 group are high. scSDAE imputed least false positive values in the CA1Pyr1 group.

To more explicitly demonstrate the difference of different loss functions, we also analyzed another dataset with simulated genes. In [48], two subclasses of cells, 351 CA1Pyr1 and 389 CA1Pyr2 cells, were identified. We added five artificial genes to the normalized expression matrix. The artificial genes in CA1Pyr1 cells were generated by normal distribution N(0, 0.1), while the artificial genes in CA1Pyr2 cells were generated by normal distribution N(2, 0.1). Then we randomly down-sampled the five genes and obtained simulated datasets containing 10% to 90% (step: 10%) zeros, respectively. The zeros of the artificial genes in CA1Pyr1 cells can be seen as real zeros, which should not be imputed into big values; however, the zeros of the artificial genes in CA1Pyr2 cells should be imputed. To test the imputation accuracy of scSDAE, SDAE, and SDAE0, we calculated the mean absolute error (MAE) between the imputed values and the original values of the artificial genes from three aspects: total MAE, CA1Pyr1 group MAE, CA1Pyr2 group MAE (Table S3). From the table, we can see that all methods reduced the MAE after imputation, and scSDAE imputed the least false positive values in the CA1Pyr1 group. scSDAE with default parameters consistently obtained smaller MAE than SDAE in all groups and all sparsity levels. SDAE0 induced severe false signals by imputing zeros in the CA1Pyr1 group (also shown in Figure 6), although for the CA1Pyr2 group, SDAE0 gained smaller MAE. We also included scSDAE (α=0.1) for comparison. scSDAE with smaller α imputed the missing data in the CA1Pyr2 group better, in the meanwhile, induced more false signals in the CA1Pyr1 group, but performed better than SDAE0 and SDAE on total MAE in most cases. In summary, scSDAE tries to balance the sparse penalty on zero values and the reconstruction loss on non-zero values through α, in order to avoid false signals and to impute the technical zeros to some extent. 

## 5. Conclusions

In this study, we proposed a deep learning-based method, scSDAE, to impute the missing values in single-cell RNA-seq data. We utilized stacked denoising autoencoders that can capture the complex nonlinear relationships among data. The loss of traditional SDAE is MSE on all the features, and the loss of SDAE0 only contains the MSE loss of the non-missing features. Different from SDAE and SDAE0, we adopted a mixture loss of MSE on non-missing values and sparse penalty on zero values, which incorporated the information of the zeros and distinguished them from the observed values. We also adopted layer-wise pretraining to train the stacked denoising autoencoders to improve the fitting ability of the model. 

We compared scSDAE with MAGIC, scImpute and DCA, as well as other autoencoder-based methods, on both simulation study and real data analysis. scSDAE not only obtained better performance on recovering the true values influenced by technical variations and doing clustering analysis, but also induced less false signals. The results not only showed that the SDAE framework could work well on single-cell RNA-seq data imputation comparing with traditional methods, but also demonstrated that the mixture loss proposed in this paper fitted the problem well by comparison with SDAE and SDAE0. Nevertheless, DCA and SAUCIE which impute and denoise all the values including non-zero ones often induced severe false signals. In addition, DCA without pretraining performed not well enough on small-size datasets for clustering, while scSDAE performed well on several small-size datasets, which demonstrates the power of layer-wise pretraining of the deep neural networks when the data size is small. 

Overall, we suggest people take an imputation step before analyzing scRNA-seq data since the severe technical zeros obstruct the analysis of it and the results in the paper showed that scSDAE could help recover the true values. To impute scRNA-seq data, there are two things whereof people need special caution. First, people should avoid over-imputing and introducing false signals, especially when using methods like MAGIC, DCA, and SAUCIE. Second, procedures to improve the model fitting of deep neural networks need to be taken when using them to impute small-size datasets.

## Figures and Tables

**Table 1:** The mean and standard deviation of the CMDs between the original and the dropout/imputed matrix.

Zero Rate	Dropout	scSDAE	scImpute	MAGIC	DCA	SAUCIE
50%	0.295	0.094	0.604	0.430	0.546	0.298
	(0.003)	(0.023)	(0.027)	(0.006)	(0.173)	(0.016)
60%	0.318	0.101	0.796	0.447	0.642	0.305
	(0.004)	(0.013)	(0.009)	(0.004)	(0.235)	(0.018)
70%	0.360	0.109	0.847	0.849	0.685	0.300
	(0.005)	(0.021)	(0.024)	(0.013)	(0.285)	(0.013)
80%	0.503	0.136	0.830	0.990	0.790	0.325
	(0.009)	(0.019)	(0.020)	(0.000)	(0.244)	(0.037)
90%	0.717	0.193	0.932	0.991	0.991	0.516
	(0.009)	(0.039)	(0.007)	(0.000)	(0.000)	(0.039)

**Table 2:** ARIs of the clustering results of five datasets imputed by different methods.

Dataset	Raw	scSDAE	scImpute	MAGIC	SDAE0	SDAE	DCA
Kolodziejczyk	0.368	0.831	0.668	0.525	0.413	0.552	0.008
Pollen	0.829	0.932	0.824	0.657	0.635	0.824	0.411
Zeisel	0.747	0.794	0.776	0.696	0.736	0.764	0.702
Lake	0.591	0.687	0.446	0.556	0.301	0.491	0.017
Patel	0.857	0.864	0.663	0.848	0.857	0.857	0.084

## Supplementary Materials

The following are available online at https://www.mdpi.com/2073-4425/11/5/532/s1. Figure S1: Heatmaps of expression data show scSDAE best recovers gene expression affected by simulated missing values. Figure S2: Scatter plots of gene expression trajectory of gene *F26E4.5*. Figure S3: False gene–gene correlations induced by single-cell imputation methods. Figure S4: Accuracy of detecting differentially expressed (DE) genes in splatter simulations before and after imputation. Figure S5: (a) PCA plots after Linnorm normalization for the RNAmix_CEL-seq2 dataset., (b) Heatmaps of Pearson correlation coefficients of samples after SAUCIE imputation in the CEL-seq2 RNA mixture dataset. Figure S6: Boxplot of the Pearson correlations of the protein–RNA pairs in CITE-seq data. Figures S7–S13: Feature plots of the cells in CITE-seq data representing expression levels of protein, and corresponding RNA of gene CD3(*CD3E*), CD19(*CD19*), CD4(*CD4*); CD8(*CD8A*), CD56(*NCAM1*), CD16(*FCGR3A*), and CD14(*CD14*). Figure S14–S20: Two-dimensional UMAP visualization of the Pollen dataset after imputation of raw data, scSDAE, scImpute, MAGIC, SDAE0, SDAE, and DCA. Table S1: Pairwise Pearson correlations of the bulk data and the imputed data. Table S2: Mean absolute error (MAE) of the bulk data and the imputed data for methods which can work directly on log-normalized data. Table S3: Mean absolute error (MAE) between imputed values and the original values.

## Appendix A

Availability of Data and Material

The code to implement scSDAE with Python is available at https://github.com/klovbe/scSDAE. Bulk microarray gene expression of developing *C. elegans* embryos was downloaded from the supplementary material of [30]. RNA mixture dataset was download form https://github.com/LuyiTian/sc_mixology. Spike-in RNA dataset [28] was obtained from the GEO accession GSE79750. CITE-seq dataset [43] was obtained from the GEO accession GSE100866. Kolodziejczyk data [33] was obtained from the ArrayExpress accession E-MTAB-2600. Pollen [34] data was obtained from the SRA accession SRP041736. Zeisel data [35] was obtained from the GEO accession GSE60361. Lake data [36] was obtained from https://www.scap-t.org/ with accession phs000833.v3.p1. Patel [37] was obtained from the GEO accession GSE57872.
