# Supplementary material for: Sparsity-Penalized Stacked Denoising Autoencoders for Imputing Single-Cell RNA-seq Data
Source: Genes (Basel). 2020 May 11;11(5):532. doi: 10.3390/genes11050532 (PMC7291078; doi:10.3390/genes11050532)
Supplement: Supplementary file 1 [file genes-11-00532-s001.pdf]

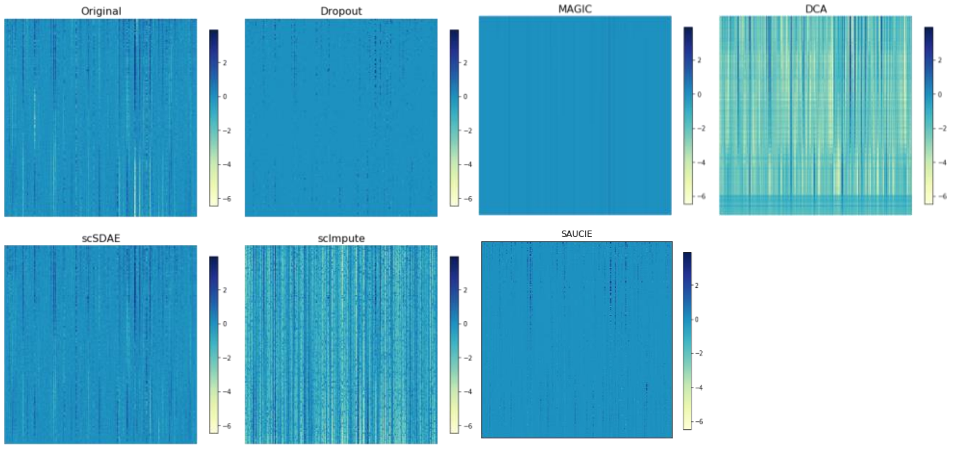

Figure S1. Heatmaps of expression data show scSDAE best recovers gene expression affected by simulated missing values. Heatmaps of the 200 most variable genes from the original, missing values introduced and imputed expression data are shown. The horizontal axis represents the developmental time of the samples. The vertical axis stands for the genes.

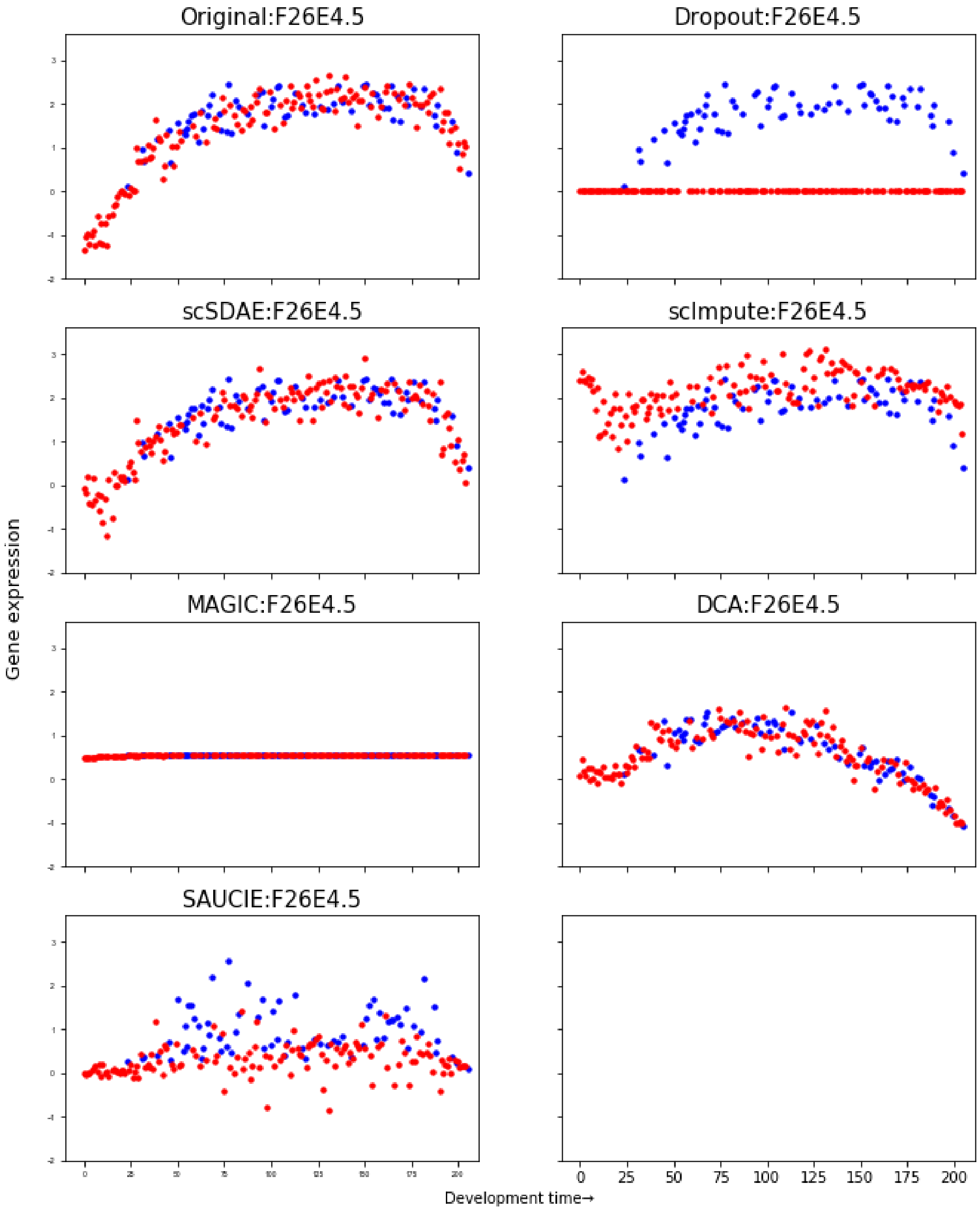

Figure S2. Scatter plots of gene expression trajectory of gene F26E4.5.

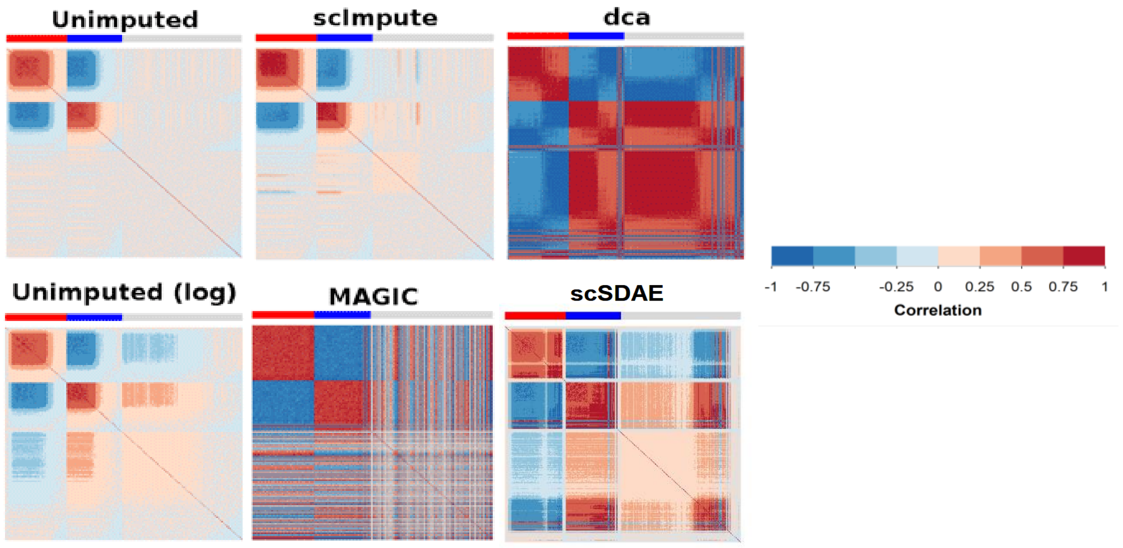

(a)

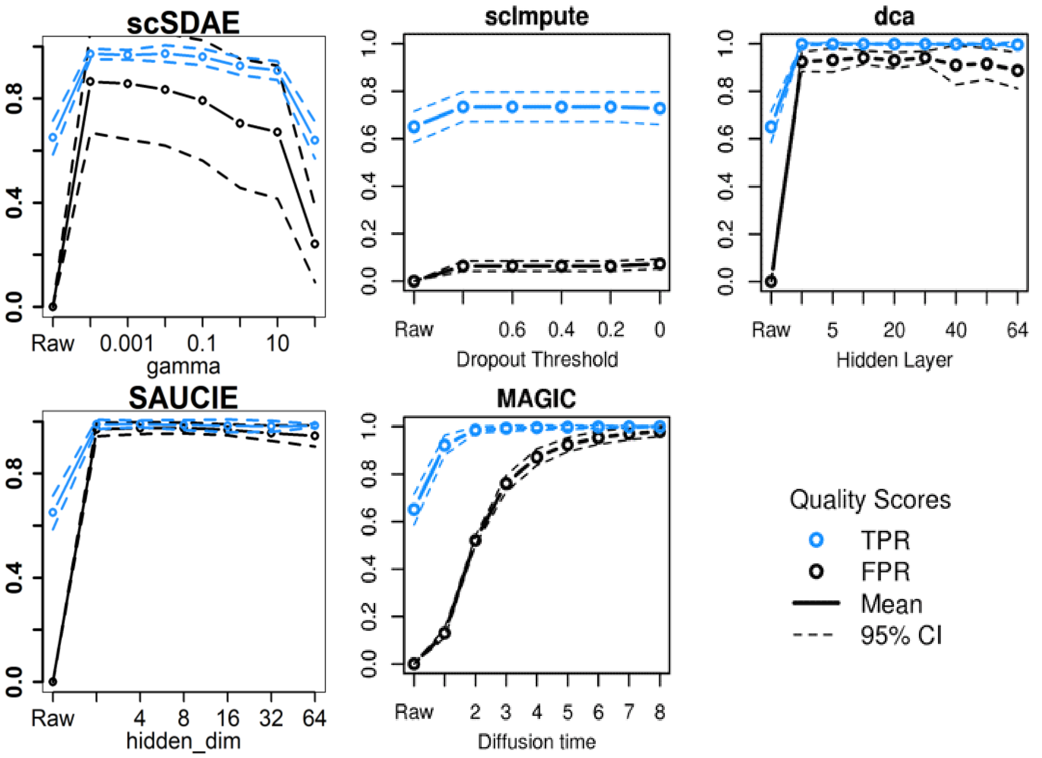

(b)

Figure S3. False gene-gene correlations induced by single-cell imputation methods. (a) Gene-gene correlation heatmaps after imputation by different imputation methods. Colored bars indicate genes highly expressed (red) or lowly expressed (blue) in one cell population vs the other, or genes not differentially expressed between the populations (grey). Genes are ordered left to right by DE direction then by expression level (high to low). (b) False positive and true positive gene-gene correlations ($p < 0.05$ Bonferroni multiple testing correction) as imputation parameters are changed. "Raw" indicates results for unimputed data. Dashed lines are 95% CIs based on 10 replicates. Figures for methods except scSDAE and SAUCIE were adapted from [30].

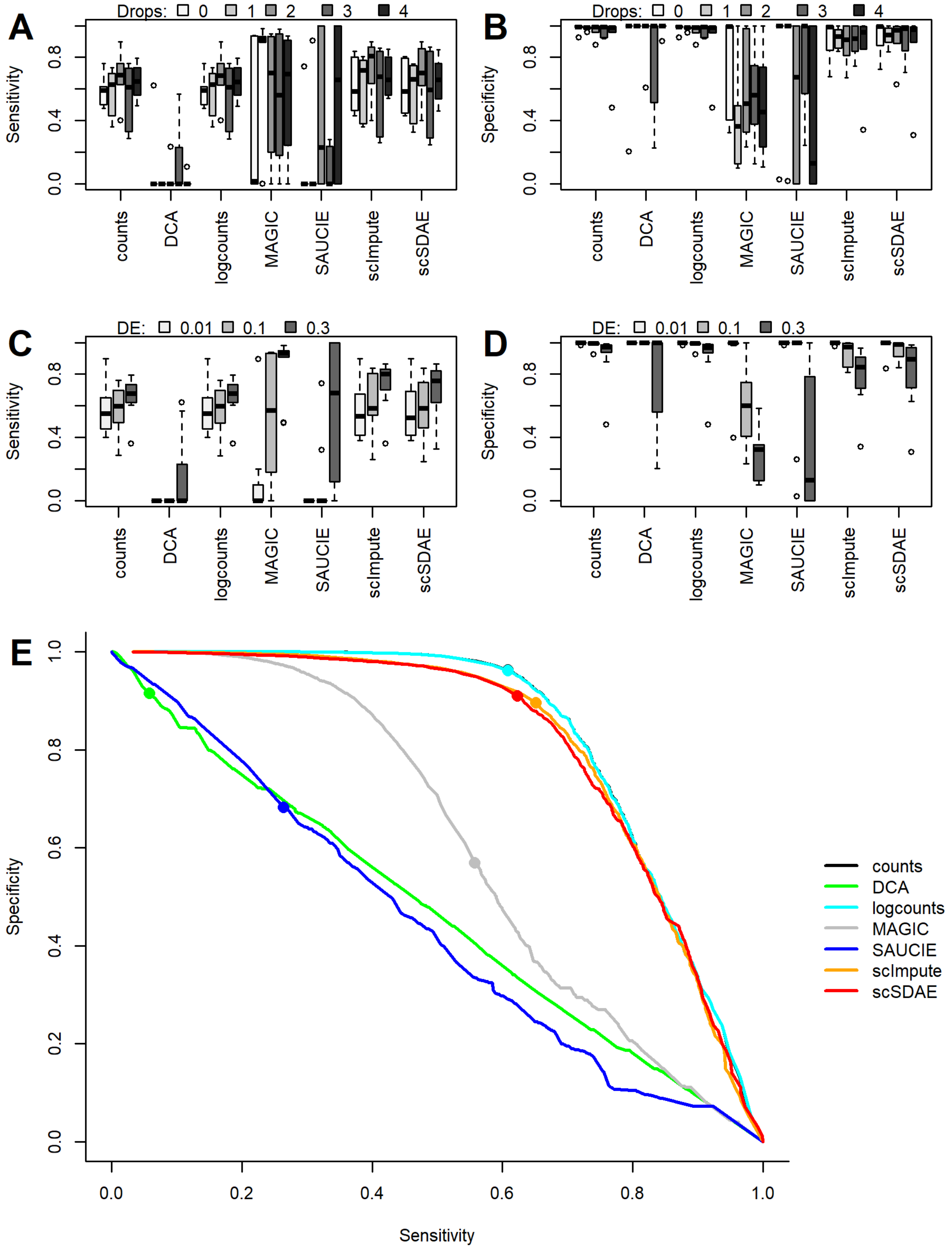

Figure S4. Accuracy of detecting differentially expressed (DE) genes in splatter simulations before and after imputation. (A & B) Zero inflation in our setting didn't show significant influence on DE detection. (C & D) Strong true signals (high proportion of DE genes) increased sensitivity and decreased specificity. (E) ROC curves across all simulations, solid dots indicate 5% FDR. Counts were normalized by total library size prior to testing DE.

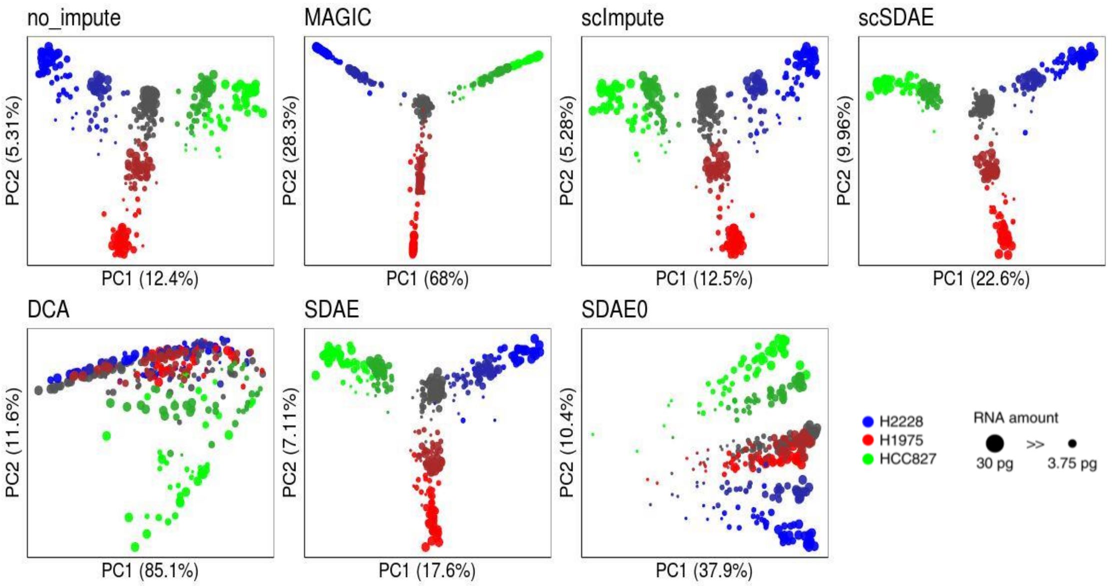

(a)

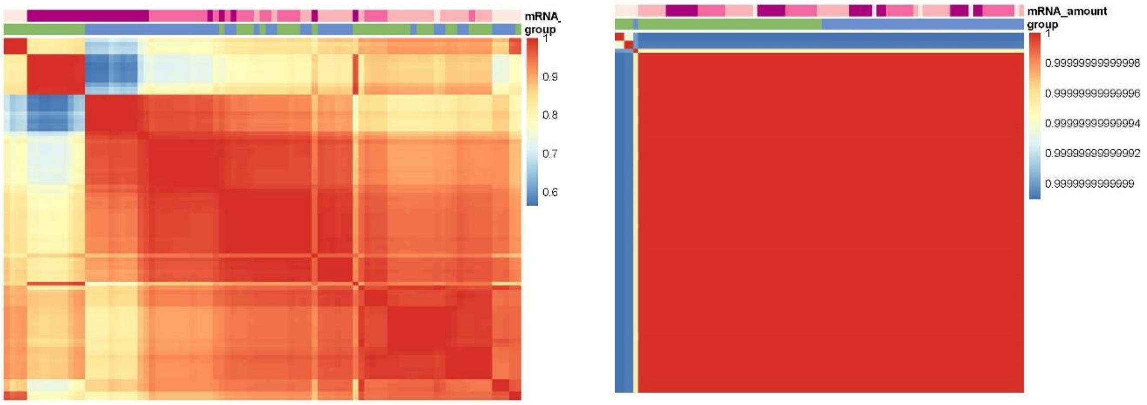

(b)

Figure S5. (a) PCA plots after Linnorm normalization and various imputation by different methods using the RNAmix_CEL-seq2 dataset (n= 340). Percentage variation explained by each principal component is included in the respective axis labels. (b) Heatmaps of Pearson correlation coefficients of samples after SAUCIE imputation in the CEL-seq2 RNA mixture dataset that have pure H2228 (n= 45) or HCC827 (n= 44) RNA obtained from TMM normalization (left) and logCPM normalization (right).

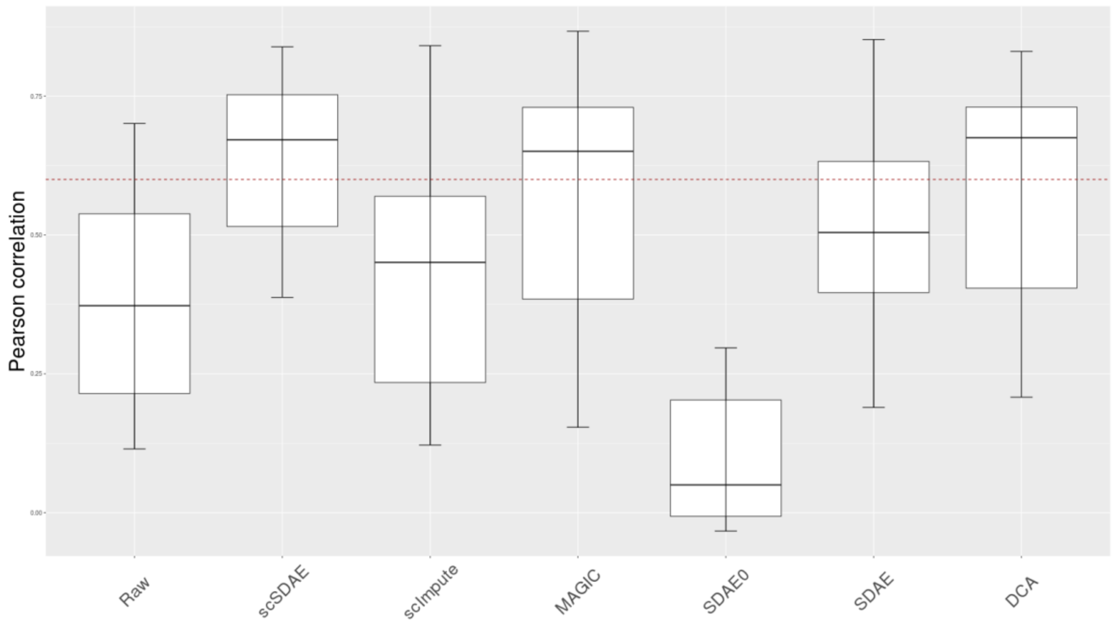

Figure S6. Boxplot of the Pearson correlations of the protein-RNA pairs in CITE-seq data.

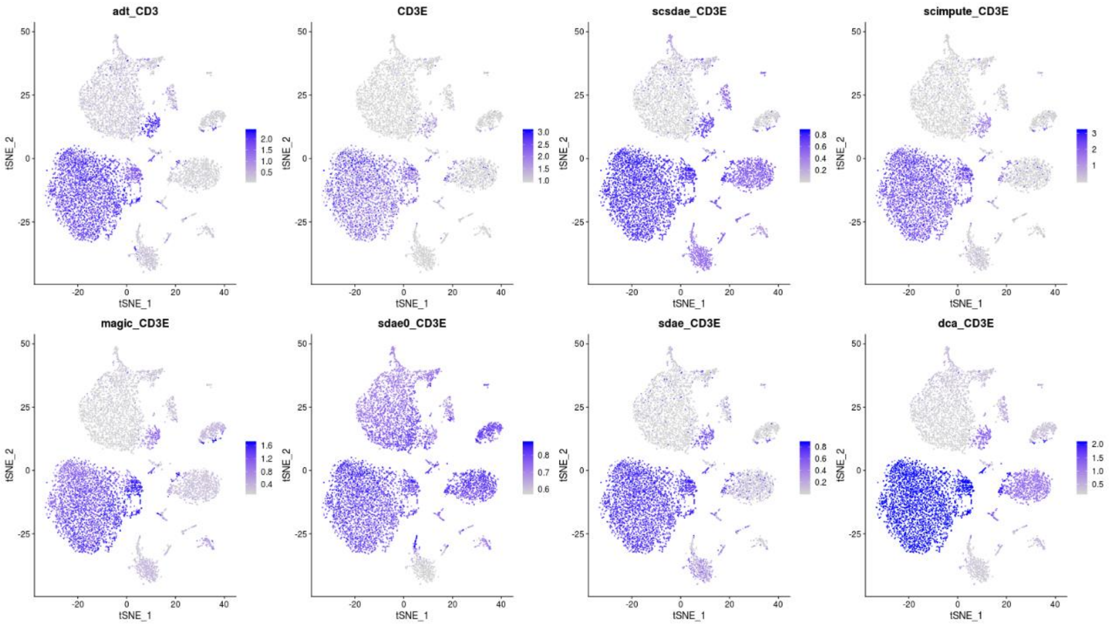

Figure S7. Featureplot of the cells in CITE-seq data representing expression levels of protein and corresponding RNA of gene CD3.

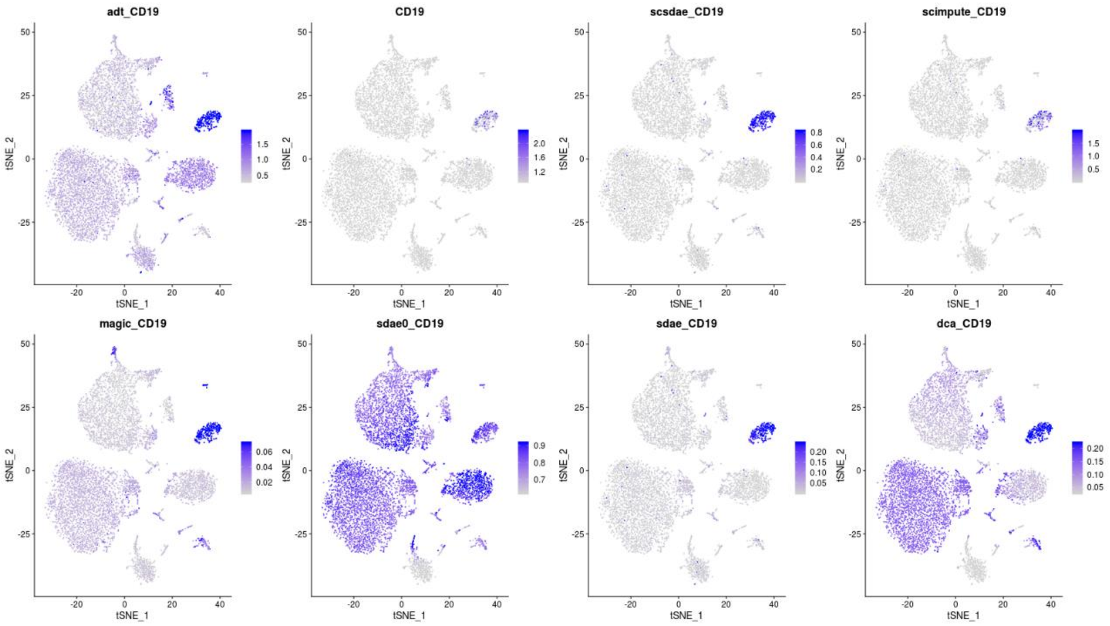

Figure S8. Featureplot of the cells in CITE-seq data representing expression levels of protein and corresponding RNA of gene CD19.

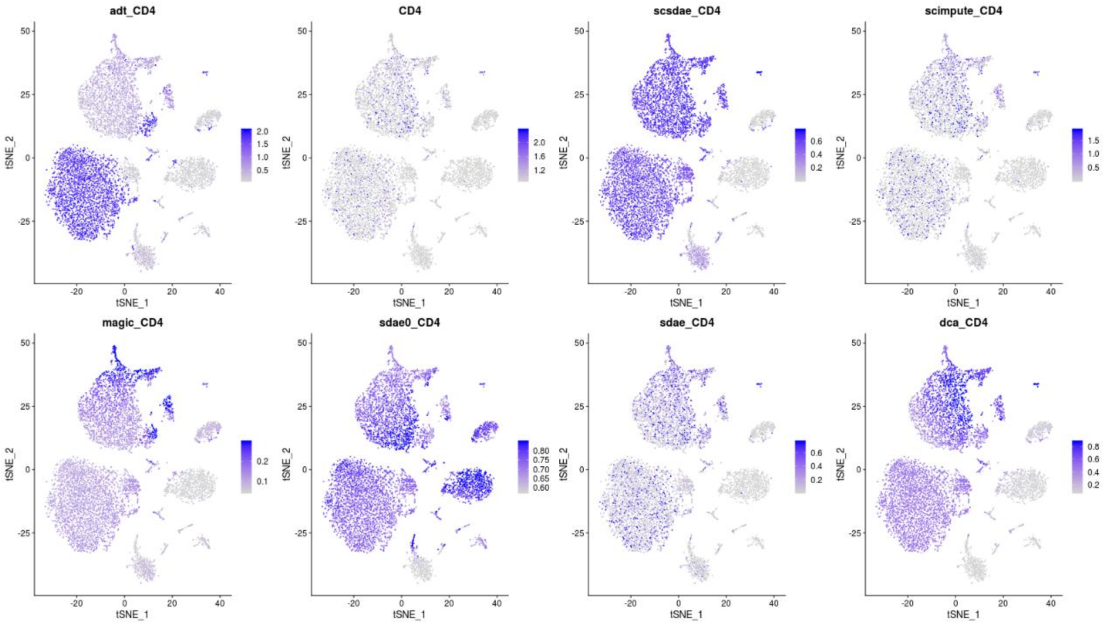

Figure S9. Featureplot of the cells in CITE-seq data representing expression levels of protein and corresponding RNA of gene CD4.

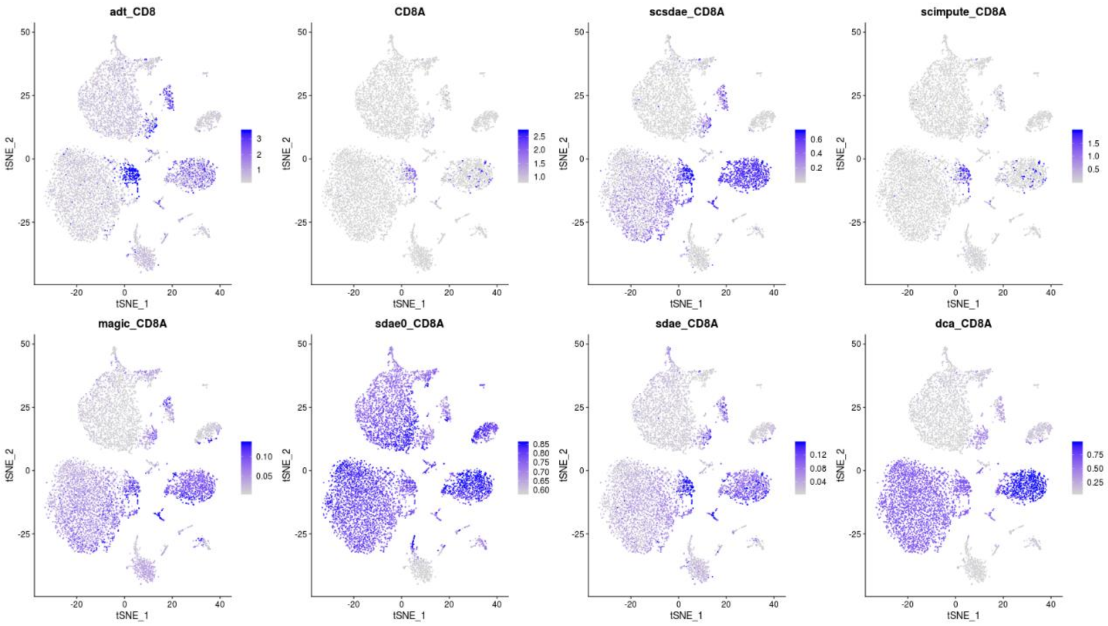

Figure S10. Featureplot of the cells in CITE-seq data representing expression levels of protein and corresponding RNA of gene CD8.

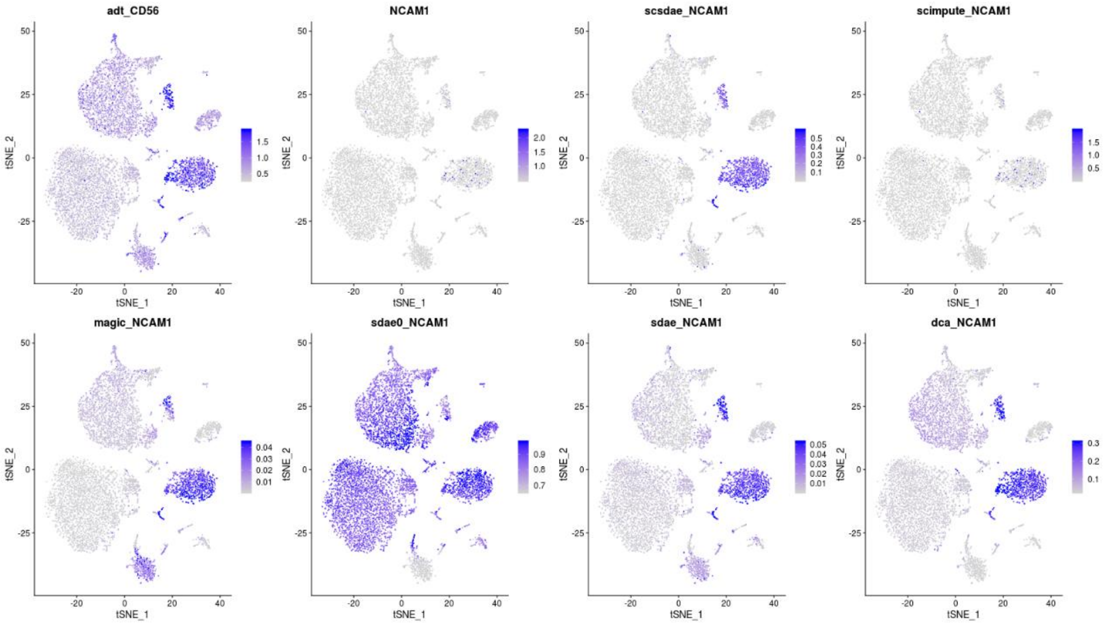

Figure S11. Featureplot of the cells in CITE-seq data representing expression levels of protein and corresponding RNA of gene CD56.

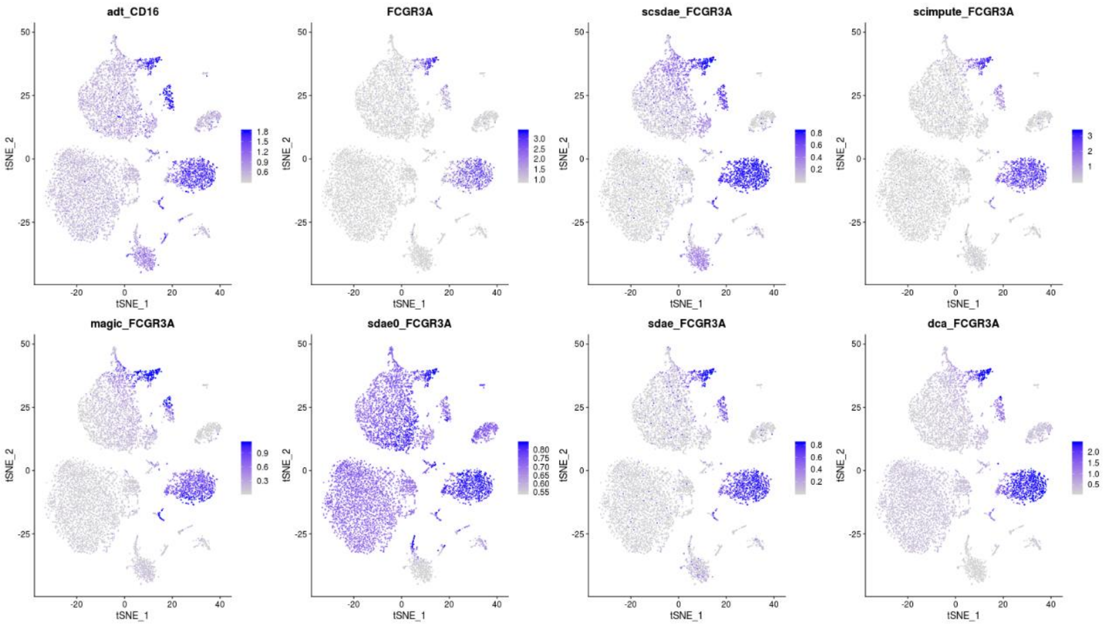

Figure S12. Featureplot of the cells in CITE-seq data representing expression levels of protein and corresponding RNA of gene CD16.

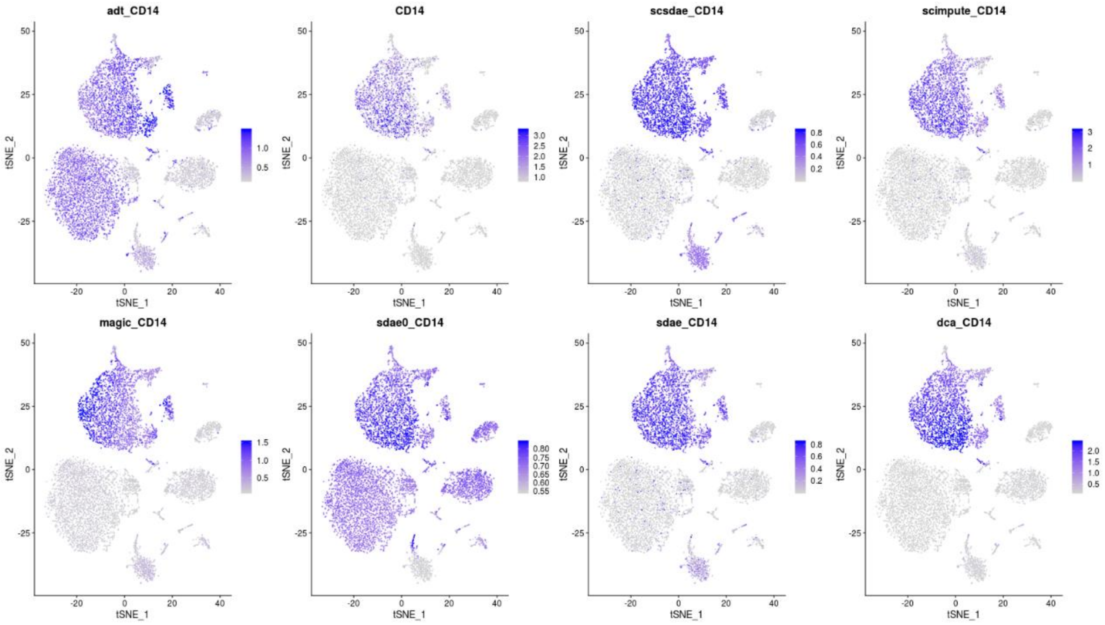

Figure S13. Featureplot of the cells in CITE-seq data representing expression levels of protein and corresponding RNA of gene CD14.

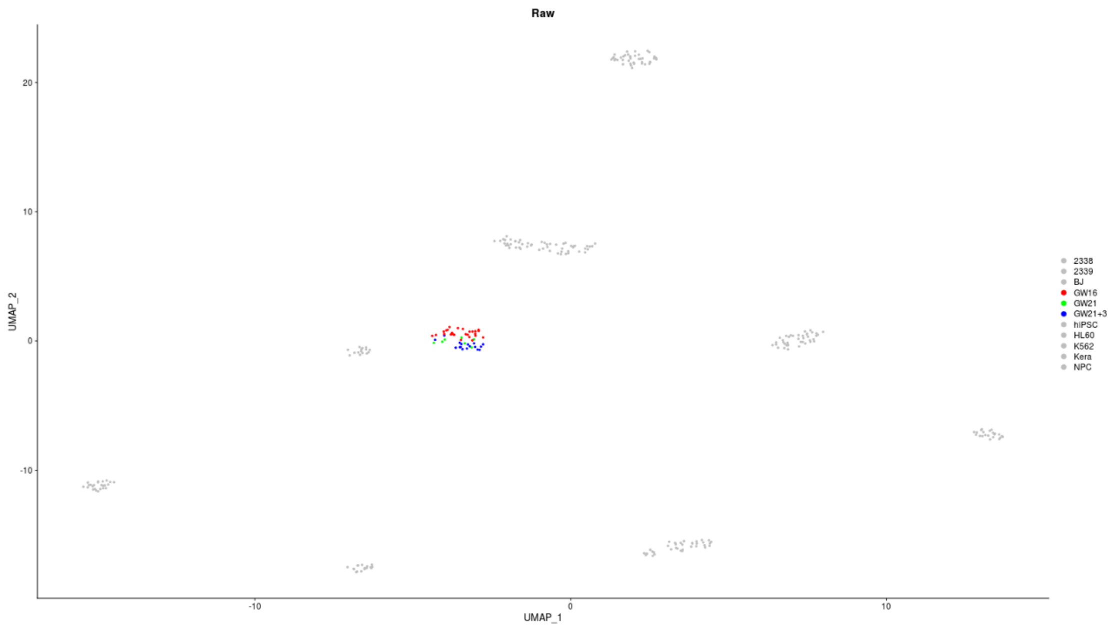

Figure S14. Two-dimensional UMAP visualization of the Pollen dataset. Colored points represent single cells from the germinal zone of human cortex at gestational week (GW16), primary cells from the cortex at GW21 and GW21 cells further cultured for 3 weeks (GW21+3).

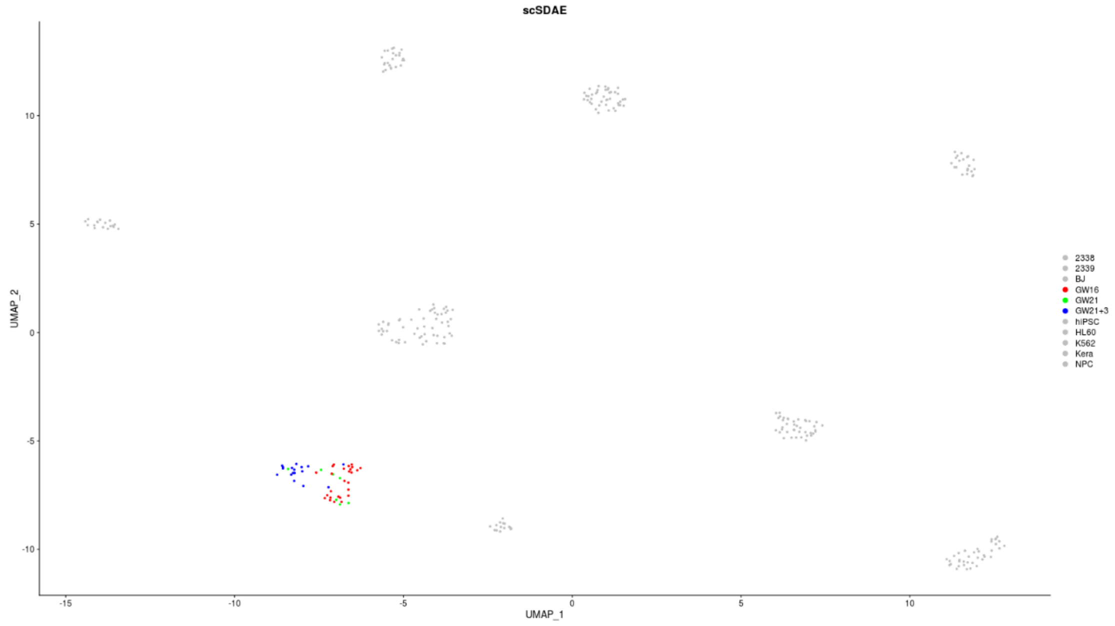

Figure S15. Two-dimensional UMAP visualization of the Pollen dataset after imputation of scSDAE. Colored points represent single cells from the germinal zone of human cortex at gestational week (GW16), primary cells from the cortex at GW21 and GW21 cells further cultured for 3 weeks (GW21+3).

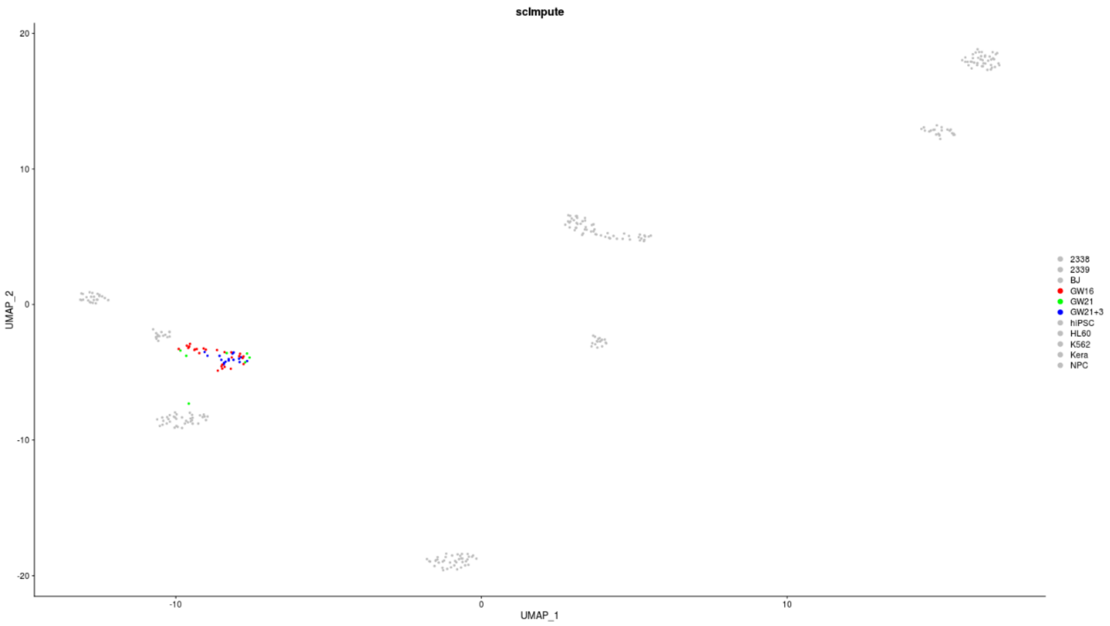

Figure S16. Two-dimensional UMAP visualization of the Pollen dataset after imputation of scImpute. Colored points represent single cells from the germinal zone of human cortex at gestational week (GW16), primary cells from the cortex at GW21 and GW21 cells further cultured for 3 weeks (GW21+3).

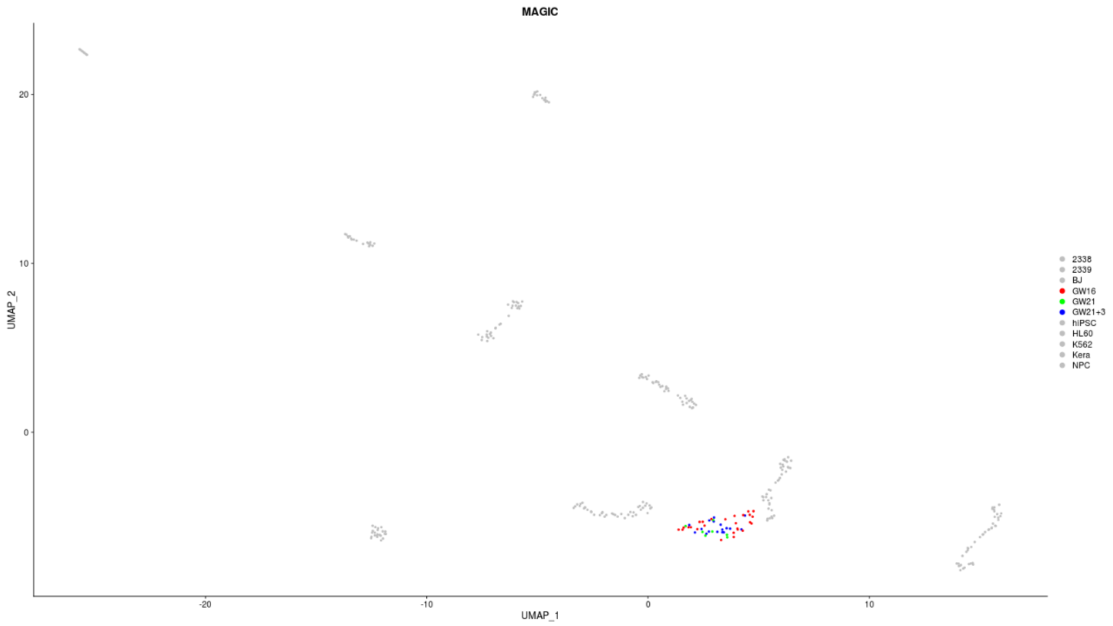

Figure S17. Two-dimensional UMAP visualization of the Pollen dataset after imputation of MAGIC. Colored points represent single cells from the germinal zone of human cortex at gestational week (GW16), primary cells from the cortex at GW21 and GW21 cells further cultured for 3 weeks (GW21+3).

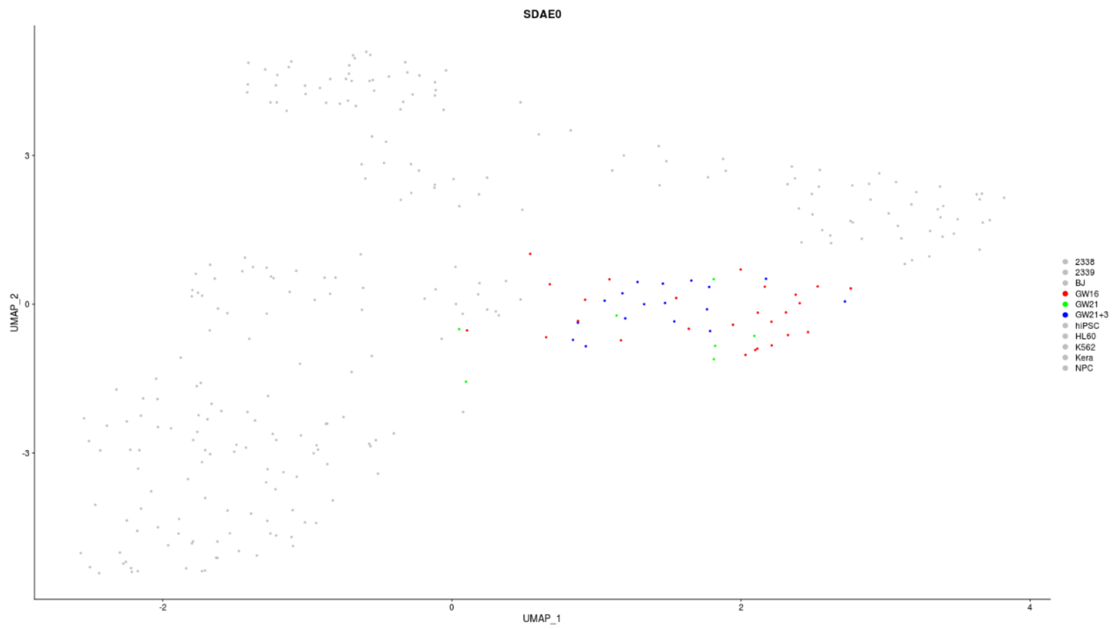

Figure S18. Two-dimensional UMAP visualization of the Pollen dataset after imputation of SDAE0. Colored points represent single cells from the germinal zone of human cortex at gestational week (GW16), primary cells from the cortex at GW21 and GW21 cells further cultured for 3 weeks (GW21+3).

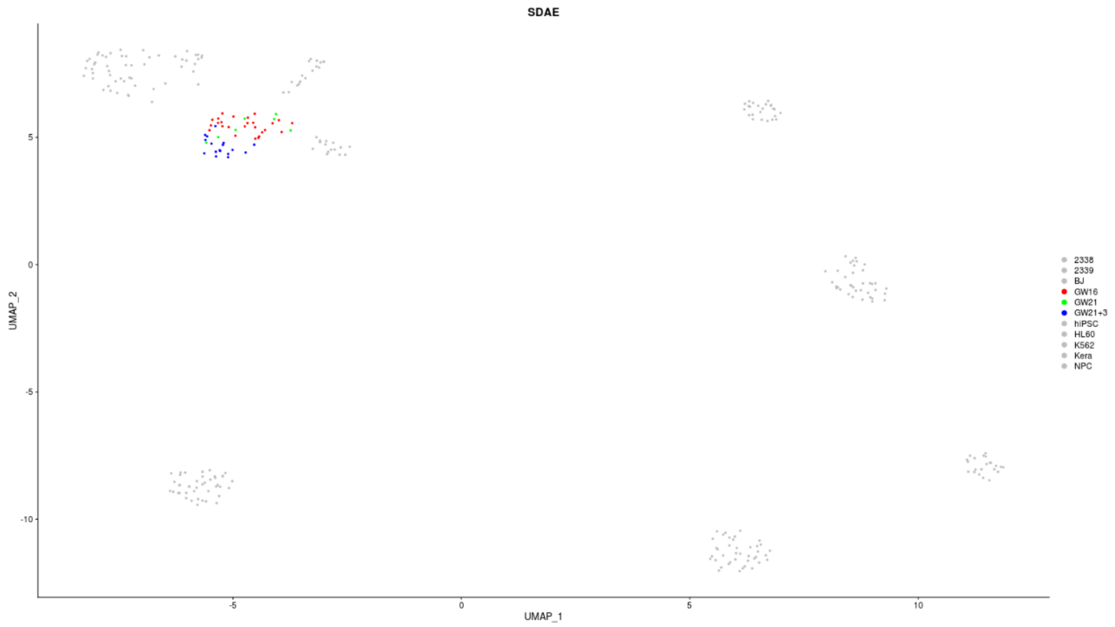

Figure S19. Two-dimensional UMAP visualization of the Pollen dataset after imputation of SDAE. Colored points represent single cells from the germinal zone of human cortex at gestational week (GW16), primary cells from the cortex at GW21 and GW21 cells further cultured for 3 weeks (GW21+3).

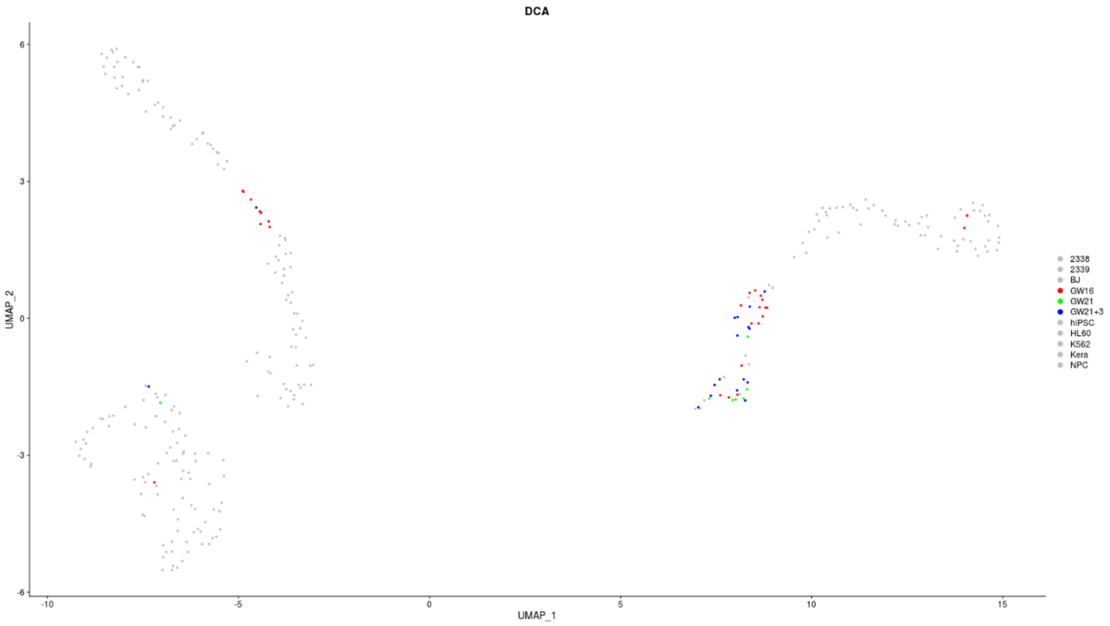

Figure S20. Two-dimensional UMAP visualization of the Pollen dataset after imputation of DCA. Colored points represent single cells from the germinal zone of human cortex at gestational week (GW16), primary cells from the cortex at GW21 and GW21 cells further cultured for 3 weeks (GW21+3).

Table S1. Pairwise Pearson correlations of the bulk data and the imputed data.

Zero Rate	Raw	scSDAE	scImpute	MAGIC	DCA	SAUCIE
50%	0.815 (0.000)	0.844 (0.004)	0.756 (0.001)	0.775 (0.000)	0.673 (0.006)	0.791 (0.002)
60%	0.796 (0.000)	0.834 (0.005)	0.717 (0.002)	0.765 (0.000)	0.682 (0.010)	0.780 (0.001)
70%	0.680 (0.000)	0.814 (0.003)	0.703 (0.002)	0.722 (0.001)	0.669 (0.026)	0.750 (0.003)
80%	0.480 (0.001)	0.782 (0.004)	0.700 (0.002)	0.648 (0.001)	0.639 (0.053)	0.611 (0.015)
90%	0.299 (0.001)	0.758 (0.002)	0.605 (0.002)	0.646 (0.001)	0.664 (0.027)	0.370 (0.007)

Table S2. Mean absolute error (MAE) between the bulk data and the imputed data for methods which can work directly on log-normalized data.

Zero Rate	Drouput	scSDAE	MAGIC	SAUCIE
50%	0.248	0.182	0.350	0.326
60%	0.272	0.202	0.355	0.335
70%	0.344	0.237	0.418	0.377
80%	0.438	0.288	0.500	0.455
90%	0.507	0.324	0.5334	0.514

Table S3. Mean absolute error (MAE) between imputed values and the original values

Dropout rate(%)	Data state/ Imputation method	Total MAE	CA1Pyr1 group MAE	CA1Pyr2 group MAE
90	Downsampling	0.956	0.037	1.785
90	scSDAE	0.226	0.064	0.372
90	scSDAE(α = 0.1)	0.169	0.203	0.138
90	SDAE	0.284	0.128	0.426
90	SDAE0	0.175	0.270	0.089
80	Downsampling	0.857	0.033	1.602
80	scSDAE	0.216	0.076	0.343
80	scSDAE(α = 0.1)	0.154	0.181	0.129
80	SDAE	0.277	0.136	0.405
80	SDAE0	0.178	0.246	0.117
70	Downsampling	0.743	0.028	1.388
70	scSDAE	0.221	0.072	0.354
70	scSDAE(α = 0.1)	0.157	0.180	0.136
70	SDAE	0.281	0.143	0.406
70	SDAE0	0.176	0.280	0.082
60	Downsampling	0.651	0.025	1.217
60	scSDAE	0.212	0.067	0.343
60	scSDAE(α = 0.1)	0.160	0.177	0.145
60	SDAE	0.280	0.134	0.411
60	SDAE0	0.189	0.295	0.092
50	Downsampling	0.537	0.020	1.003
50	scSDAE	0.214	0.080	0.334
50	scSDAE(α = 0.1)	0.145	0.163	0.129
50	SDAE	0.283	0.131	0.420
50	SDAE0	0.169	0.272	0.076
40	Downsampling	0.421	0.016	0.786
40	scSDAE	0.231	0.090	0.359
40	scSDAE(α = 0.1)	0.177	0.215	0.142
40	SDAE	0.281	0.146	0.403
40	SDAE0	0.168	0.253	0.092
30	Downsampling	0.334	0.011	0.626
30	scSDAE	0.147	0.073	0.214
30	scSDAE(α = 0.1)	0.152	0.227	0.084
30	SDAE	0.193	0.144	0.237
30	SDAE0	0.143	0.242	0.053
20	Downsampling	0.216	0.008	0.404
20	scSDAE	0.099	0.094	0.104
20	scSDAE(α = 0.1)	0.112	0.174	0.057
20	SDAE	0.137	0.144	0.130
20	SDAE0	0.122	0.216	0.037
10	Downsampling	0.112	0.004	0.210

10	scSDAE	0.075	0.113	0.042
10	scSDAE(α = 0.1)	0.110	0.202	0.028
10	SDAE	0.099	0.147	0.056
10	SDAE0	0.112	0.212	0.023
